# Salmonella enterica Serovar Typhimurium Alters the Extracellular Proteome of Macrophages and Leads to the Production of Proinflammatory Exosomes

**DOI:** 10.1128/IAI.00386-17

**Published:** 2018-01-22

**Authors:** Winnie W. Hui, Kamil Hercik, Sayali Belsare, Navatha Alugubelly, Beata Clapp, Carlos Rinaldi, Mariola J. Edelmann

**Affiliations:** a Department of Microbiology and Cell Science, University of Florida, Gainesville, Florida, USA; b Department of Basic Sciences, College of Veterinary Medicine, Mississippi State University, Mississippi State, Mississippi, USA; c J. Crayton Pruitt Family Department of Biomedical Engineering, University of Florida, Gainesville, Florida, USA; d Department of Infectious Diseases and Immunology, University of Florida, Gainesville, Florida, USA; e Department of Chemical Engineering, University of Florida, Gainesville, Florida, USA; University of California, Davis

**Keywords:** Salmonella enterica serovar Typhimurium, macrophage, proteomics, ubiquitin, OTUB1, extracellular vesicles, exosomes, *S*. Typhimurium, ubiquitination

## Abstract

Salmonella enterica serovar Typhimurium is a Gram-negative bacterium, which can invade and survive within macrophages. Pathogenic salmonellae induce the secretion of specific cytokines from these phagocytic cells and interfere with the host secretory pathways. In this study, we describe the extracellular proteome of human macrophages infected with *S*. Typhimurium, followed by analysis of canonical pathways of proteins isolated from the extracellular milieu. We demonstrate that some of the proteins secreted by macrophages upon *S*. Typhimurium infection are released via exosomes. Moreover, we show that infected macrophages produce CD63^+^ and CD9^+^ subpopulations of exosomes at 2 h postinfection. Exosomes derived from infected macrophages trigger the Toll-like receptor 4-dependent release of tumor necrosis factor alpha (TNF-α) from naive macrophages and dendritic cells, but they also stimulate secretion of such cytokines as RANTES, IL-1ra, MIP-2, CXCL1, MCP-1, sICAM-1, GM-CSF, and G-CSF. Proinflammatory effects of exosomes are partially attributed to lipopolysaccharide, which is encapsulated within exosomes. In summary, we show for the first time that proinflammatory exosomes are formed in the early phase of macrophage infection with *S*. Typhimurium and that they can be used to transfer cargo to naive cells, thereby leading to their stimulation.

## INTRODUCTION

Secreted proteins represent a significant protein subset, acting as messengers in intercellular communication. Salmonella enterica serovar Typhimurium is a Gram-negative bacterium critical from the food safety standpoint (1). Throughout various stages of *S*. Typhimurium infection, differential regulation of protein secretion orchestrates immune responses. Excellent examples of secretory proteins are cytokines such as interleukin-4 (IL-4) and IL-10, which inhibit immune defense against *S*. Typhimurium, but also IL-1α and interferon gamma (IFN-γ), which are protective (2). Other cytokines, such as IL-1β and IL-18, can lead to an upregulation of inflammasome (3), and they are activated by Salmonella pathogenicity island 1 (SPI-1) proteins (4). However, altered secretion of other proteins is also expected to follow infection since *S*. Typhimurium can interfere with the host endosomal pathway, which is tightly associated with protein secretion (5, 6).

Shotgun proteomics enables identification and quantification of multiple proteins concurrently, and it has been utilized to reveal changes in the host protein pathways in several infection models (7). We describe here an extracellular proteome of human THP-1-derived macrophages infected with *S*. Typhimurium at early stages of infection. We also define the canonical pathways of these extracellular proteins and provide evidence that exosomes are involved in secretion of specific protein cargo from infected cells, which include a human deubiquitinating enzyme, OTUB1, identified by secretomics. Finally, we show that these exosomes activate naive macrophages, which release proinflammatory cytokines upon treatment with exosomes derived from infected macrophages, but not when treated with exosomes derived from uninfected macrophages. Moreover, exosomes derived from infected dendritic cells (DCs) also stimulate the release of proinflammatory cytokines in naive DCs. These data show that an activation of immune cells during infection can be achieved via exosomes and therefore suggest that exosomes are organelles involved in the innate immune responses.

## RESULTS

### Cytotoxicity in macrophages is not significantly altered by *S*. Typhimurium infection at 2 hpi.

**FIG 1:**
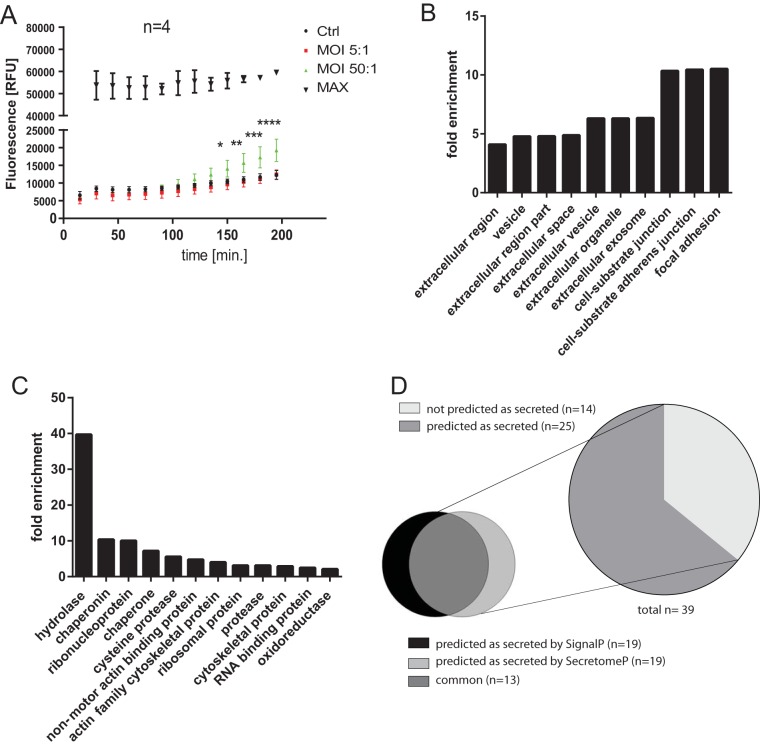
(A) Cytotoxicity in macrophages infected with *S*. Typhimurium. THP-1 macrophages were infected with *S*. Typhimurium (MOI of 5:1 or 50:1) or left uninfected (*n* = 4). A CellTox Green cytotoxicity assay (Promega) was used, which enables measurement of changes in cytotoxicity at several time points (0 to 195 min postinfection). Alternatively, cells were lysed, to obtain 100% cytotoxicity measurement (MAX). Two-way ANOVA test was used for statistical analysis (GraphPad Prism). Means and standard deviations are shown for each sample. The *P* values are shown indicated for comparisons between control (uninfected) cells and cells infected with *S*. Typhimurium at an MOI of 50:1. For an MOI of 5:1, none of the results were significant. The *P* values are indicated by asterisks as follows: *, *P* ≤ 0.05; **, *P* ≤ 0.01; ***, *P* ≤ 0.001; and ****, *P* ≤ 0.0001. (B and C) GO terms of extracellular proteins identified by proteomics. All proteins obtained from cell culture medium of uninfected and *S*. Typhimurium-infected THP-1 macrophages were analyzed by label-free mass spectrometry, and their GO terms were analyzed by the PANTHER overrepresentation test. The GO molecular function and GO cellular component were both analyzed, Homo sapiens was used as a reference gene list for the fold enrichment analysis, and a Bonferroni correction for multiple testing was used in each case. The top GO terms were chosen in terms of the statistical significance (the smallest *P* value), and the fold enrichment for each term is shown. (D) *Ab initio* and signal peptide predictions of extracellular proteins with abundance significantly affected by *S*. Typhimurium infection. SignalP and SecretomeP were used to analyze the extracellular proteins with abundance affected by *S*. Typhimurium infection (Table 1).

We used a cytotoxicity assay (CellTox Green cytotoxicity assay; Promega, USA) to show that the cell cytotoxicity was not significant at 1.5 and 2 h postinfection (hpi). This cytotoxicity assay enabled measurement of changes in cytotoxicity at several time points (Fig. 1A). At 90 and 120 min of infection the changes in cytotoxicity were not significant, but at 150 min onward the cytotoxicity was significantly increased upon infection with *S*. Typhimurium at a multiplicity of infection (MOI) of 50:1. At an MOI of 5:1, the cytotoxicity has not been substantially increased even at 195 min postinfection. Successful infection was confirmed by using a gentamicin protection assay (Fig. S1 and data not shown). LDH assay was also performed at 90 min postinfection (1.5 hpi), and it did not indicate increased cytotoxicity (data not shown). At 1.5 hpi, macrophages were successfully infected, and yet the cell membranes were still intact, avoiding nonspecific escape of cytosolic proteins.

### Extracellular proteome of *S*. Typhimurium-infected and uninfected THP-1 macrophages.

Our goal was to describe changes in the extracellular proteome of THP-1 macrophages infected with *S*. Typhimurium. Macrophages were infected with *S*. Typhimurium for 1.5 h, followed by extraction of proteins from cell culture supernatants and their proteomic analysis (Table 1; see also Table S1 in the supplemental material). Gene Ontology (GO) analysis was used to investigate the subcellular localization of identified proteins, which were focal adhesions, cell-substrate junctions, cell-substrate adherens junctions, exosomes, extracellular space, and extracellular regions (Fig. 1B). GO analysis also mapped the molecular functions (Fig. 1C) of identified proteins, which were annotated as hydrolases, chaperonins, ribonucleoproteins, chaperones, cysteine proteases, actin-binding proteins, ribosomal proteins, and proteases. Next, we focused our analysis on the proteins of which the secretion was significantly increased or decreased upon *S*. Typhimurium infection, based on significantly altered fold change (>2) and *P* values (*P* < 0.5) determined by Fisher exact test (Table 1; see also Table S1 in the supplemental material). More than 64% of proteins in this subset were predicted as secreted (Fig. 1D), either due to the presence of an N-terminal signal peptide (48.7%) or because they were predicted by SecretomeP as targets of noncanonical secretion (48.7% [8]).

**TABLE 1:**
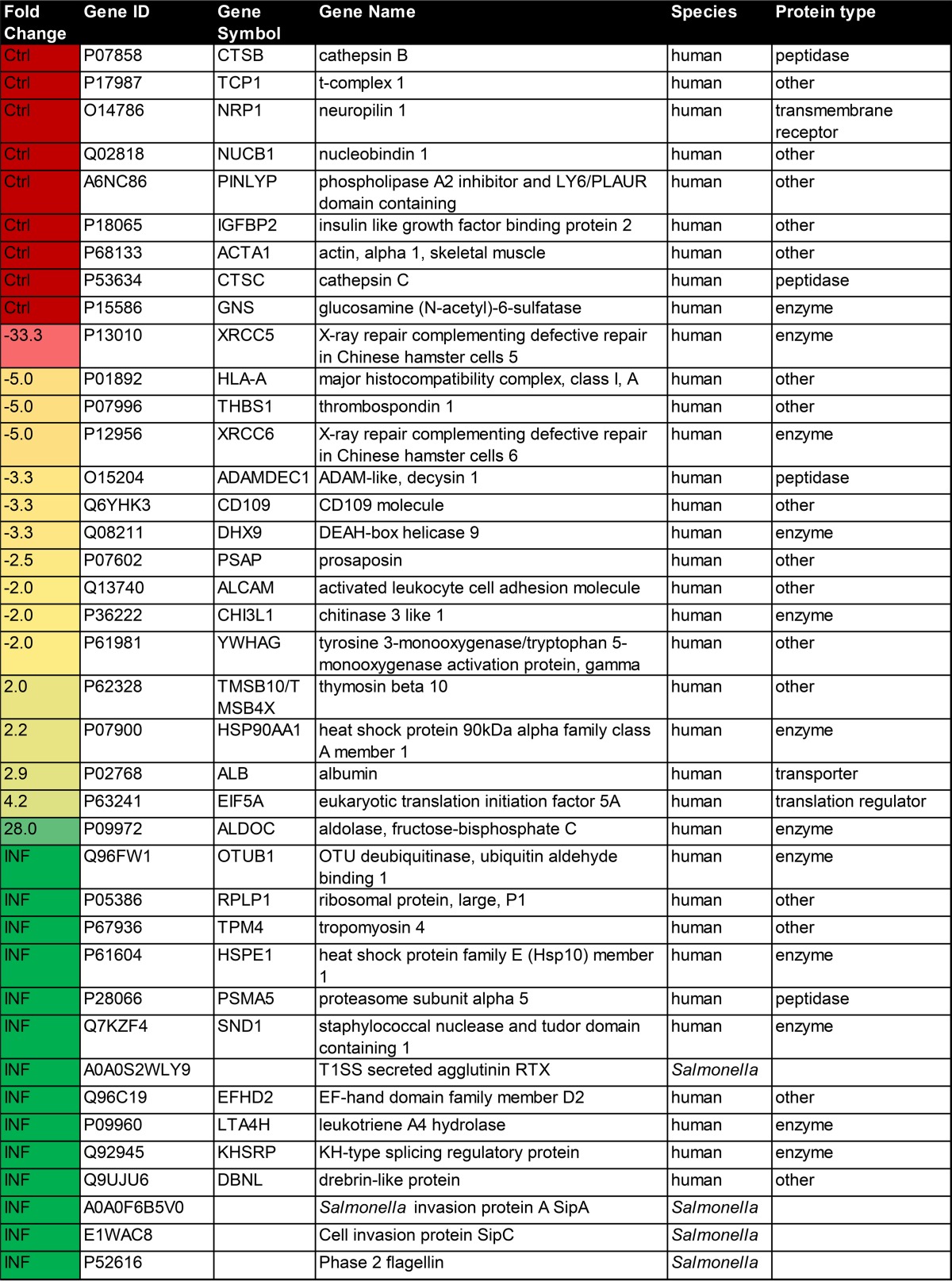
Extracellular proteins of THP-1 macrophages with abundance affected by *S*. Typhimurium infection^*a*^

a Fold change indicates up- or downregulation of extracellular proteins from infected cells in comparison to uninfected cells. UniProt ID, gene (protein) name, and species are shown. Gene symbol and protein types are exported from the IPA software (if known). Additional information is available in Table S1 in the supplemental material. The minimum protein identification probability for shown proteins is 95%. The *P* values were calculated using the Fisher exact test and spectral counts of two independent biological replicates, where a minimum *P* value was 0.05. SEQUEST identifications required delta Cn scores of greater than 0.2 and XCorr scores of greater than 1.2, 1.9, 2.3, and 2.6 for singly, doubly, triply, and quadruply charged peptides, respectively. The reported peptide FDR was 0.03%, and the protein FDR was 0.2%. Proteins identified only in control or only in infected samples are indicated as Ctrl and INF, respectively.

### Pathway modeling and molecular function analysis of extracellular proteins modulated during Salmonella infection.

**FIG 2:**
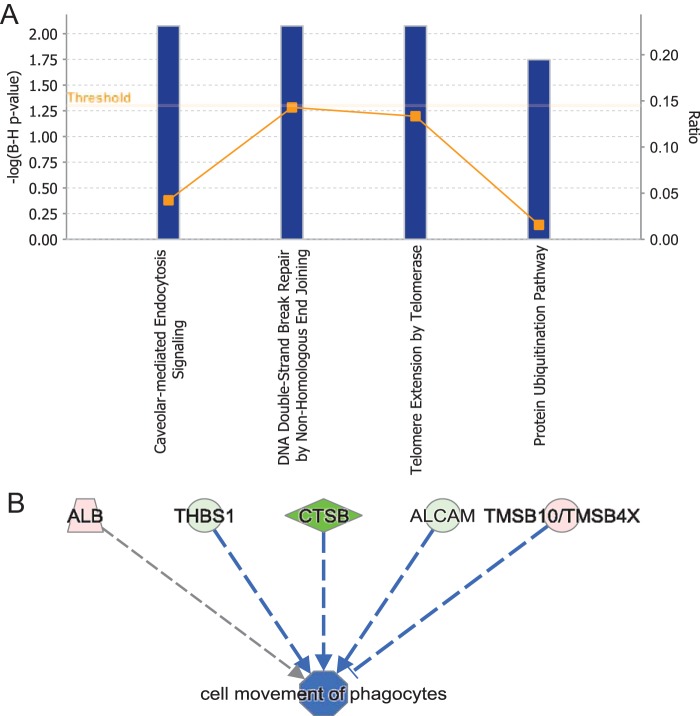
Canonical pathways of extracellular proteins with abundance affected by *S*. Typhimurium infection. IPA software was used to analyze extracellular proteins with abundance regulated by *S*. Typhimurium infection. (A) Canonical pathways were analyzed by the Fisher exact test with Hochberg-Bonferroni multiple testing corrections. The calculated significance represents the probability of association of proteins with the canonical pathway by random chance, and the −log of this *P* value is shown on the *y* axis of each graph. The square points connected by a line represent the ratio, which indicates the number of genes in a pathway from the data set divided by the total number of genes in the pathway (a reference gene list). (B) Cell movement of phagocytes was identified by IPA as one of the top downregulated functions of identified extracellular proteins with abundance affected by *S*. Typhimurium infection.

Ingenuity Pathway Analysis (IPA) software was used to map the extracellular proteins affected by *S*. Typhimurium infection (Table 1) onto physiological pathways and identify protein networks (Fig. 2). The canonical pathway analysis indicated that the extracellular proteins affected by *S*. Typhimurium infection are involved in such pathways as caveola-mediated endocytosis signaling, protein ubiquitination, and others (Fig. 2A). Moreover, proteins with decreased secretion (cathepsin B [CTSB], activated leukocyte cell adhesion molecule [ALCAM], and thrombospondin 1 [THBS1]) and increased secretion (thymosin β10 [TSMB10/TSMB4X]) upon *S*. Typhimurium infection were shown to contribute to an inhibition of cell movement of phagocytes (Fig. 2B).

**FIG 3:**
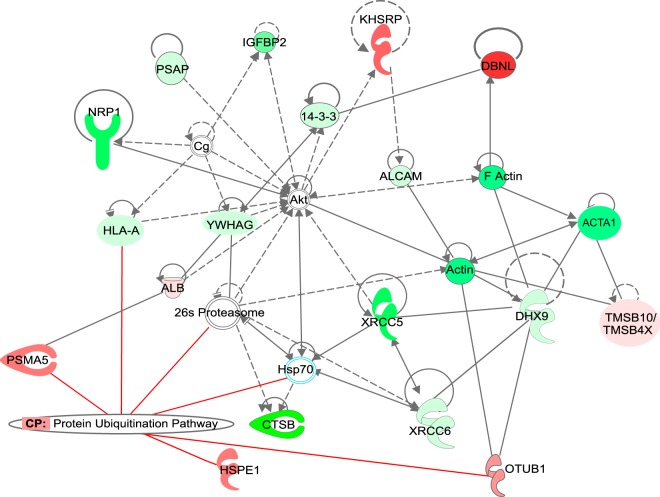
Top protein network identified among extracellular proteins with significantly altered abundance upon *S*. Typhimurium infection. The top protein network was overlaid by Ingenuity Pathway Analysis (IPA) software with canonical pathways to indicate major processes regulated by the identified extracellular proteins.

The most significant protein network (Fig. 3) was overlaid with the protein ubiquitination pathway as one of the top canonical pathways controlled by extracellular proteins (increased secretion of PSMA5, OTUB1, or HSPE1, or decreased secretion of HLA-A) differentially regulated in *S*. Typhimurium infection. Identification of this network indicates that proteins controlling ubiquitination might be present extracellularly during early stages of *S*. Typhimurium infection. Ubiquitination is known to be significantly involved in regulating the host response to *S*. Typhimurium, and several pathogen-encoded virulence factors modulate this pathway (9). However, the extracellular presence of ubiquitin processing enzymes during *S*. Typhimurium infection has never been demonstrated. We show that *S*. Typhimurium infection leads to an increased secretion of such proteins such as OTU deubiquitinase 1 (OTUB1), proteasome subunit α5 (PSMA5), EF-hand domain family member D2 (EFHD2), leukotriene A4 hydrolase, heat shock protein family E member 1 (HSPE1), or drebrin-like protein (DBNL).

### OTUB1 is secreted by macrophages infected with *S*. Typhimurium.

**FIG 4:**
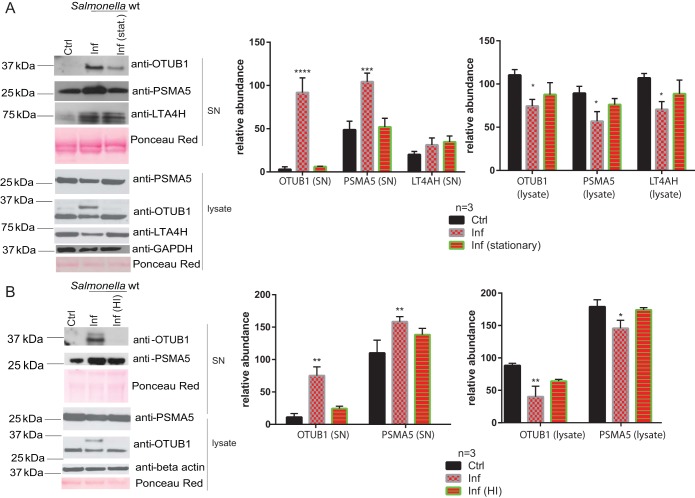
*S*. Typhimurium contributes to the differential expression of extracellular proteins of infected macrophages. (A) THP-1 macrophages were infected (or not, control [ctrl]) for 90 min with *S*. Typhimurium grown to either exponential (Inf) or stationary phase (Inf [stat.]), after which the cell culture medium and cells were separately collected. Western blotting was used to confirm the abundances of OTUB1, PSMA5, and LTA4H (detected in the proteomics study; Table 1) in cell culture supernatants and cell lysates. ImageJ was used to quantify the pixel density of the bands (*n* = 3), and the results are displayed as graphs. A Student *t* test was used for statistical analysis. (B) THP-1 macrophages were infected for 90 min with *S*. Typhimurium (Inf) or heat-inactivated (Inf [HI]) *S*. Typhimurium. Cell culture supernatants and cell lysates were collected and analyzed as described above. *P* values are indicated as follows: *, *P* ≤ 0.05; **, *P* ≤ 0.01; ***, *P* ≤ 0.001; and ****, *P* ≤ 0.0001.

**FIG 5:**
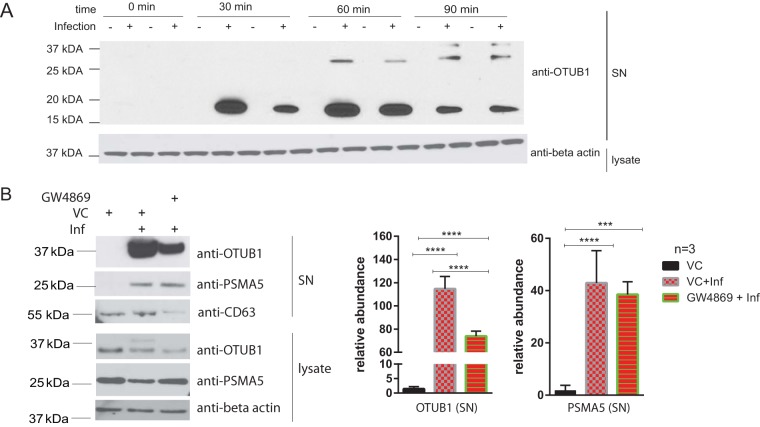
(A) THP-1 macrophages were infected or left uninfected for 0, 30, 60, and 90 min with *S*. Typhimurium. Cell culture medium was collected at each time point in duplicates and analyzed by Western blotting with anti-OTUB1 and anti-β-actin antibodies (a loading control for cell lysate). (B) THP-1 macrophages were treated with GW4896 inhibitor (5 μM) or an equivalent volume of DMSO (vehicle control [VS]) for 90 min before infection. Cells were infected (Inf) for 90 min with *S*. Typhimurium at an MOI of 50:1 or left uninfected. CD63 (exosome marker), OTUB1, and PSMA5 were detected in cell culture supernatant (SN), whereas OTUB1, PSMA5, and β-actin (a loading control) were detected in cell lysate by Western blotting. ImageJ was used to quantify the pixel density of the bands (*n* = 3). A Student *t* test was used for statistical analysis. The results are displayed as relative abundances on graphs. *P* values are indicated as follows: *, *P* ≤ 0.05; **, *P* ≤ 0.01; ***, *P* ≤ 0.001; and ****, *P* ≤ 0.0001.

We have validated several of the differentially secreted proteins by using Western blotting (Fig. 4). When stationary-phase *S*. Typhimurium (Fig. 4A) was used to infect THP-1 macrophages, the levels of secreted OTUB1 and PSMA5 were decreased compared to cells infected with exponential-phase bacteria. Similarly, when heat-inactivated *S*. Typhimurium (Fig. 4B) was used, the levels of secreted OTUB1 and PSMA5 were also decreased in comparison to cells infected with live bacteria. An increased secretion of OTUB1 and PSMA5 corresponded to reduced intracellular protein levels of these proteins (Fig. 4), while the mRNA levels of OTUB1 were not significantly increased upon *S*. Typhimurium infection (see Fig. S2 in the supplemental material). OTUB1 deubiquitinase was chosen for further validation due to its involvement in the invasion of other Gram-negative bacteria (10, 11), and its regulatory function in protein ubiquitination (Fig. 2 and 3). OTUB1 was detected in the cell culture supernatant of infected cells from 60 min postinfection onward, with its secretion increasing with the time of infection (tested up to 120 min postinfection; Fig. 5A and data not shown). Moreover, a profiling experiment utilizing ubiquitin-specific active-site probe labeling of extracellular deubiquitinases (12) indicated that OTUB1 is active extracellularly and that there are other active deubiquitinases present in the extracellular milieu, which are regulated upon *S*. Typhimurium infection (see Fig. S3 in the supplemental material).

Since OTUB1 sequence does not contain a secretory motif (13), we next tested whether OTUB1 is released via the exosomal pathway. We used neutral sphingomyelinase 2 (nSMase2) inhibitor GW4869, which inhibits exosome release from multivesicular bodies in an ESCRT-independent pathway (14). *S*. Typhimurium-infected cells treated with GW4869 released significantly less OTUB1 in comparison to vehicle-treated cells. Moreover, CD63 levels in the supernatants of GW4869-treated infected cells were reduced compared to vehicle control (Fig. 5B). CD63 is a tetraspanin present on exosomes, and it serves as an exosome marker, next to other tetraspanins such as CD9 but also the HSP70 heat shock protein (15). Extracellular levels of PSMA5 upon infection were not significantly affected by GW4869 treatment, suggesting that PSMA5 might not be a target of secretion via exosomes. This experiment suggests that OTUB1 is released by exosomes in a nSMase2-dependent pathway.

### OTUB1 constitutes a cargo of exosomes released from macrophages upon *S*. Typhimurium infection.

**FIG 6:**
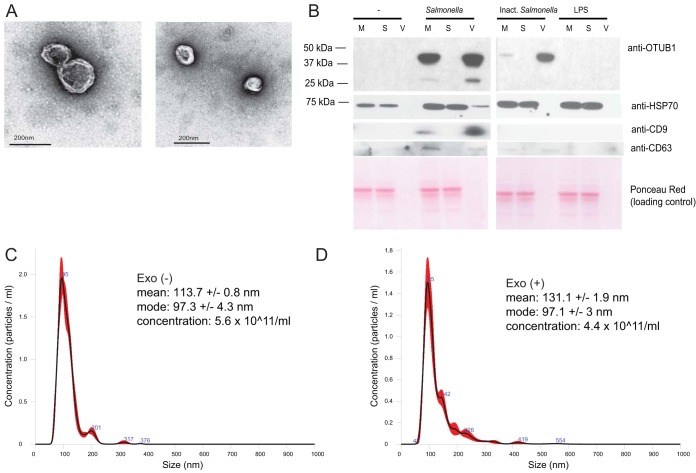
Exosomes produced by macrophages infected with *S*. Typhimurium at 2 hpi. THP-1 macrophages were left uninfected or infected with wild-type viable or paraformaldehyde-inactivated *S*. Typhimurium (Inact. Salmonella). Alternatively, the cells were treated with *S*. Typhimurium-derived LPS. Cell culture supernatants were collected 2 hpi and subjected to serial centrifugation to isolate exosomes. (A) Exosomes derived from *S*. Typhimurium-infected macrophages were visualized by transmission electron microscopy. (B) Cell culture medium (M), vesicle-free supernatant (S), and the extracellular vesicle fraction (V) were analyzed by Western blotting to show the presence of OTUB1, HSP70, CD63, and CD9 in the exosomes. Ponceau Red was used to stain protein material, and HSP70, CD63, and CD9 were used as exosome markers. (C and D) Size distribution of exosomes produced by THP-1 macrophages at 2 hpi. THP-1 macrophages were left uninfected (C) or were infected with wild-type *S*. Typhimurium (D). Exosomes were purified at 2 hpi from the cell culture supernatant by serial centrifugation. The mean and mode sizes of the exosomes (nm) and the concentrations of exosomes (number of particles/ml) were recorded by NanoSight. Exo(+), exosomes derived from infected macrophages; Exo(−), exosomes derived from uninfected macrophages.

We hypothesized that OTUB1 is released from infected cells via exosomes. We extended infection time to 2 hpi to obtain a significant number of vesicles at early stages of infection. We isolated extracellular vesicles (EVs) from the culture medium of THP-1 infected macrophages and analyzed them by electron microscopy (Fig. 6A). THP-1 produced EVs, which contained exosomal markers, including CD9 and CD63 tetraspanins, as well as HSP70 (Fig. 6B) (16). The identified cup-shape morphology of these vesicles, the presence of exosomal protein markers, and the size of the vesicles indicate that these EVs are exosomes. We performed Western blotting of EVs, vesicle-free supernatants remaining after EVs were pelleted (S), and culture medium containing both of these fractions (M). This analysis showed that OTUB1 is localized in EVs but not in the EV-free cell culture medium (S; Fig. 6B). Chemical inactivation of *S*. Typhimurium also led to a partial increase in secretion of OTUB1, but lipopolysaccharide (LPS) treatment alone did not have this effect (Fig. 6B). This finding further strengthens our hypothesis that OTUB1 is targeted to exosomes upon *S*. Typhimurium infection.

Nanoparticle tracking analysis indicated that exosomes produced by macrophages have mode diameters of 97.3 nm ± 4.3 nm (exosomes derived from uninfected cells; Fig. 6C) and 97.1 ± 3 nm (exosomes derived from infected cells; Fig. 6D). Overall, we confirmed that the size of exosomes derived from THP-1 cells is within the expected range of ∼100 nm (16). The concentration of exosomes was measured to estimate an average number of exosomes produced per cell. Approximately 4.4 × 10^11^ exosomes are produced from 50 × 10^6^ infected THP-1 cells (Fig. 6C and D), indicating that in average ∼8,800 exosomes are released from one infected cell. Exosomes obtained from uninfected and infected (2 hpi) mouse RAW 264.7 macrophages, primary bone marrow-derived dendritic cells (BMDCs), and bone marrow-derived macrophages (BMDMs) also have a size consistent with the size range of exosomes (Fig. S4 in the supplemental material and data not shown).

### Exosomes derived from infected macrophages trigger tumor necrosis factor alpha (TNF-α) production in naive cells.

**FIG 7:**
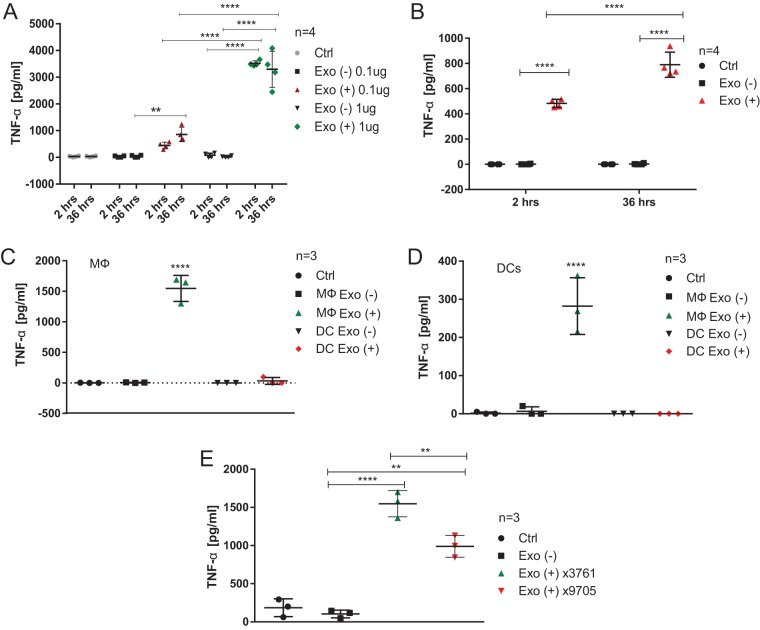
Exosomes derived from infected macrophages stimulate release of TNF-α from naive macrophages. (A) RAW 264.7 mouse macrophages were infected with wild-type *S*. Typhimurium (UK-1; MOI of 5:1) or left uninfected. Exosomes were collected from cell culture supernatants at 2 and 36 hpi and their concentration was established by BCA protein assay. Naive RAW 264.7 macrophages were treated with PBS (Ctrl) or with 0.1 or 1 μg of exosomes derived from infected and uninfected macrophages. After 24 h, the cell culture supernatant was collected, and TNF-α release was measured by ELISA. Results from four biological replicates are shown. Two-way ANOVA test was used to establish statistical significance, which was done in conjunction with multiple testing correction (Tukey). Exo(+), exosomes derived from infected macrophages; Exo(−), exosomes derived from uninfected macrophages. (B) Mouse BMDMs were treated with 1 μg of exosomes derived from uninfected or *S*. Typhimurium-infected RAW 264.7 macrophages (2 and 36 hpi, MOI of 5:1). The cell culture supernatant was collected after 24 h, and TNF-α release was measured by ELISA. Four biological replicates are shown. One-way ANOVA test with Tukey's multiple testing correction was used to establish statistical significance. (C) Exosomes were isolated from uninfected and *S*. Typhimurium-infected BMDMs or BMDCs (2 hpi, MOI of 5:1). Naive BMDMs were treated with these exosomes or PBS (Ctrl) for 24 h, and TNF-α release was then measured by ELISA. The results of three biological replicates are shown. One-way ANOVA test with Tukey's multiple testing correction was used to establish statistical significance. (D) Exosomes were isolated from uninfected and *S*. Typhimurium-infected BMDMs or BMDCs as in panel C. Naive BMDCs were treated with these exosomes or PBS (Ctrl) for 24 h, and the TNF-α release was then measured by ELISA. The results of three biological replicates are shown. One-way ANOVA test with Tukey's multiple testing correction was used to establish statistical significance. (E) THP-1 macrophages were infected with wild-type *S*. Typhimurium (UK-1 χ3761; MOI of 5:1), infected with Δ*pagL7* Δ*pagP81*::Plpp *lpxE* Δ*lpxR9 S*. Typhimurium (UK-1 χ9705; MOI of 5:1), or left uninfected. Exosomes were purified from cell culture supernatant at 2 hpi. Naive THP-1 macrophages were treated with PBS (Ctrl) or with 1 μg of exosomes derived from infected and uninfected macrophages. After 24 h, the cell culture supernatant was collected, and TNF-α release was measured by ELISA. The results of three biological replicates are shown. One-way ANOVA test with Tukey's multiple testing correction was used to establish statistical significance. *P* values are indicated as follows: *, *P* ≤ 0.05; **, *P* ≤ 0.01; ***, *P* ≤ 0.001; and ****, *P* ≤ 0.0001.

We tested whether exosomes produced by infected and uninfected macrophages stimulate cytokine release in naive cells when exposed to these exosomes. RAW 264.7 mouse macrophages were infected (2 h and 36 h) or left uninfected, and crude exosomes were isolated from the cell culture supernatant and used to treat naive cells. Doses of 0.1 μg and 1 μg of exosomes derived from infected or uninfected cells were added to cell culture medium of naive RAW 264.7 macrophages for 24 h. TNF-α was measured in cell culture supernatants by enzyme-linked immunosorbent assay (ELISA) (Fig. 7A), indicating that exosomes generated as early as 2 hpi dramatically increase TNF-α release in treated cells. Moreover, a 1-μg dose of exosomes led to a significantly higher increase in TNF-α production compared to a 0.1-μg dose. Finally, equal doses of exosomes derived from uninfected macrophages did not trigger TNF-α production in naive cells (Fig. 7A). We verified that exosomal cargo is internalized by RAW 264.7 macrophages by labeling exosomes with Exo-Green stain (System Biosciences, Palo Alto, CA) and treating naive cells with these labeled exosomes for 2 h (see Fig. S5 in the supplemental material). Also, we showed that reduction in exosome production by using GW4869 inhibitor treatment leads to a lower production of TNF-α in *S*. Typhimurium-infected cells (2 hpi; see Fig. S6 in the supplemental material).

We subsequently confirmed that primary cells are similarly stimulated by exosomes derived from infected macrophages. Naive BMDMs were treated with a dose (1 μg) of exosomes derived either from uninfected or from *S*. Typhimurium-infected (2 and 36 hpi) RAW 264.7 macrophages, and TNF-α in cell culture supernatants was measured by ELISA. Exosomes derived from cells infected for 2 and 36 hpi induced TNF-α in naive BMDMs (Fig. 7B), but exosomes isolated 36 hpi led to a significantly higher level of TNF-α release compared to 2 hpi. Next, we tested exosomes derived from uninfected and infected (2 hpi) BMDMs and BMDCs. A dose (1 μg) of each one of these exosomes was used to treat BMDMs (Fig. 7C) or BMDCs (Fig. 7D) for 24 h, where phosphate-buffered saline (PBS) was used as an additional control (Ctrl). Exosomes derived from infected BMDMs triggered TNF-α release from naive BMDMs, whereas BMDC-derived exosomes did not have this effect (Fig. 7C). Furthermore, exosomes obtained from infected BMDMs triggered TNF-α release from naive BMDCs, but exosomes derived from infected BMDCs failed to do so at the detection level tested by ELISA (Fig. 7D). However, cytokine panel analysis revealed that low levels of TNF-α were released from BMDCs treated with exosomes derived from infected BMDCs (see Fig. S7A in the supplemental material). Other cytokines released from BMDCs treated with BMDC-derived exosomes included RANTES, MIP-1α, MIP-1β, MIP-2, CXCL1, and CXCL10, indicating that exosomes derived from infected DCs stimulate the release of select cytokines in naive DCs.

We then tested whether lipopolysaccharide (LPS) toxicity affects the proinflammatory effect of exosomes isolated from infected cells. We used *S*. Typhimurium expressing *lpxA* from Francisella tularensis (Δ*pagL7* Δ*pagP81*::Plpp *lpxE* Δ*lpxR9 S*. Typhimurium strain χ9705 [17]), which contains 1-dephosphorylated LPS, and therefore it exhibits attenuated endotoxicity in both animals and cells (17). Exosomes derived from THP-1 macrophages infected with the χ9705 *S*. Typhimurium strain induced significantly less TNF-α in naive cells than exosomes isolated from the wild type (strain χ3761)-infected macrophages. However, exosomes isolated from χ9705-infected macrophages triggered significantly more TNF-α in naive macrophages compared to exosomes derived from uninfected macrophages or PBS control (Fig. 7E).

Schorey and coworkers have shown that LPS is targeted to the vesicular pathway of macrophages upon infection with *S*. Typhimurium (18). To strengthen the hypothesis that LPS is partially responsible for the proinflammatory properties of host exosomes produced during infection, we used polymyxin B (PMB). PMB is an antibiotic that binds LPS and inhibits its proinflammatory effects on host cells (19–21). As expected, PMB treatment blocked effects of purified *S*. Typhimurium LPS on TNF-α release from RAW 264.7 macrophages completely (see Fig. S8A in the supplemental material). In contrast, TNF-α release from naive macrophages treated with exosomes was reduced by PMB, but only by ∼1.5-fold. These data indicate that effects of exosome-enclosed LPS cannot be fully counteracted by PMB. Therefore, LPS in exosomes might be only partially responsible for an increased TNF-α release from naive macrophages treated with these exosomes.

We next attempted to distinguish whether protein component or protein cargo of exosomes is relevant to their proinflammatory properties. Exosomes were lysed by using heat to release their content, similarly to a method described previously (22). Intact and heat-lysed exosomes were then treated with proteinase K (ProtK) to cleave and inactivate any protein component of vesicles. Intact exosomes, ProtK-treated exosomes, and lysed exosomes as well as lysed exosomes treated with ProtK were used to treat naive RAW 264.7 macrophages to evaluate their effect on TNF-α release. ProtK treatment of intact exosomes significantly decreased TNF-α release (see Fig. S8B in the supplemental material), indicating that this protease might cleave surface-exposed proteins of exosomes necessary for an induction of TNF-α. Alternatively, ProtK might be partially integrated into exosomes where it cleaves the protein material. Exosome lysis led to a significantly higher production of TNF-α as the contents of vesicles were released and readily available for treated naive cells, but this effect was reduced upon protease ProtK digestion. Further, lysis of these vesicles leads to a release of higher levels of endotoxin (see Fig. S8C in the supplemental material). These data suggest that exosome-enclosed LPS and protein components (see Fig. S8A to D in the supplemental material) might both be critical in stimulating TNF-α in naive macrophages.

### Exosomes elicit TLR4-dependent stimulation of TNF-α in naive macrophages.

**FIG 8:**
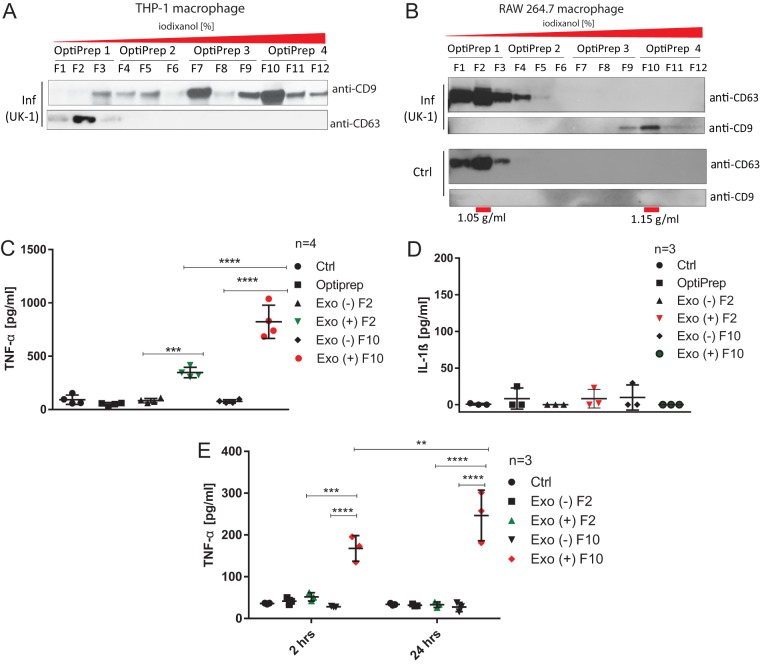
CD9^+^ and CD63^+^ subpopulations of exosomes elicit an immune response in treated macrophages and DCs. (A) THP-1 macrophages were infected with wild-type Salmonella Typhimurium (UK-1) for 2 h. Exosomes were purified from cell culture supernatants by serial centrifugation, followed by density-gradient separation using an OptiPrep discontinuous iodixanol gradient. The following concentrations (wt/vol) of iodixanol were utilized in each OptiPrep fraction: OptiPrep 1, 5% (wt/vol); OptiPrep 2, 10% (wt/vol); OptiPrep 3, 20% (wt/vol); and OptiPrep 4, 40% (wt/vol). Four subfractions from each OptiPrep fractions (F1 to F12) were collected and analyzed by Western blotting with anti-CD9 and anti-CD63 antibodies. (B) RAW 264.7 macrophages were infected (Inf) with wild-type Salmonella Typhimurium (UK-1) for 2 h or left uninfected. Exosomes were purified from cell culture supernatants as in panel A. OptiPrep fractions were collected and analyzed by Western blotting with anti-CD9 and anti-CD63 antibodies. The densities of vesicles in F2 and F10 were measured and are indicated below the graph. (C and D) Fractions (F2 and F10) of exosomes isolated from infected or uninfected RAW 264.7 macrophages (see panel B) were used to treat naive RAW 264.7 macrophages. As controls, PBS (Ctrl) and the OptiPrep solution were used. After 24 h of treatment, the concentrations of TNF-α (A) or IL-1β (D) in the cell culture supernatants were measured by ELISAs. One-way ANOVA test with Tukey's multiple testing corrections was used to establish statistical significance. Exo(+), exosomes derived from infected macrophages; Exo(−), exosomes derived from uninfected macrophages. (E) Fractions (F2 and F10) of exosomes isolated from CCS of infected or uninfected RAW 264.7 macrophages (see panel B) were used to treat naive BMDMs for 2 and 24 h. We used PBS treatment as a control (Ctrl). The concentration of TNF-α was measured by ELISA. The results of three biological replicates are shown. One-way ANOVA test with Tukey's multiple testing correction was used to establish statistical significance. *P* values are indicated as follows: *, *P* ≤ 0.05; **, *P* ≤ 0.01; ***, *P* ≤ 0.001; and ****, *P* ≤ 0.0001.

Exosomes are categorized based on the presence of several unique biomarkers such as tetraspanins (e.g., CD9, CD63, and CD81), and various exosome subpopulations have distinct contents and functions (23). We fractionated THP-1-derived exosomes (human macrophages; Fig. 8A) and RAW 264.7-derived exosomes (murine macrophage, Fig. 8B) by using a sucrose-iodixanol density gradient, which yielded distinct subpopulations of exosomes based on their density, including CD9^+^ (fractions of higher density [F10]) and CD63^+^ (fractions of lower density [F2]) exosomes. The refractive index was confirmed by density measurements, which indicated that F2 exosomes had a density of ∼1.05 g/ml, while F10 exosomes were ∼1.15 g/ml (Fig. 8B). In addition, nanoparticle tracking analysis (NTA) confirmed that the sizes of these vesicles are within the range of size expected for exosomes (see Fig. S4C to F in the supplemental material).

Next, we tested whether there is a difference in the proinflammatory properties of distinct subpopulations of exosomes, where we focused on the evaluation of F2 and F10 exosomes derived from infected (2 hpi) or uninfected RAW 264.7 macrophages (Fig. 8B). F2 and F10 exosome subpopulations derived from infected macrophages (but not from uninfected cells) triggered TNF-α release in naive RAW 264.7 cells, where F10 exosomes caused a significantly higher release of TNF-α from naive cells (Fig. 8C), but none of these exosomes induced IL-1β in murine cells (Fig. 8D). In contrast, exosomes derived from infected human THP-1 macrophages triggered TNF-α but also IL-1β (and not IL-10) release from naive THP-1 macrophages (see Fig. S9 in the supplemental material). This difference is likely caused by the fact that RAW 264.7 macrophages have a deficiency in apoptosis-associated speck-like protein containing a C-terminal caspase recruitment domain (ASC), which is one of the inflammasome components, thereby disabling processing and release of IL-1β in RAW 264.7 cells (24). Moreover, F10-positive exosomes derived from infected RAW 264.7 cells triggered a time-dependent TNF-α production in BMDMs treated with these exosomes for 2 and 24 h (Fig. 8E), inducing an elongated cell morphology in these naive BMDMs (see Fig. S10 in the supplemental material).

**FIG 9:**
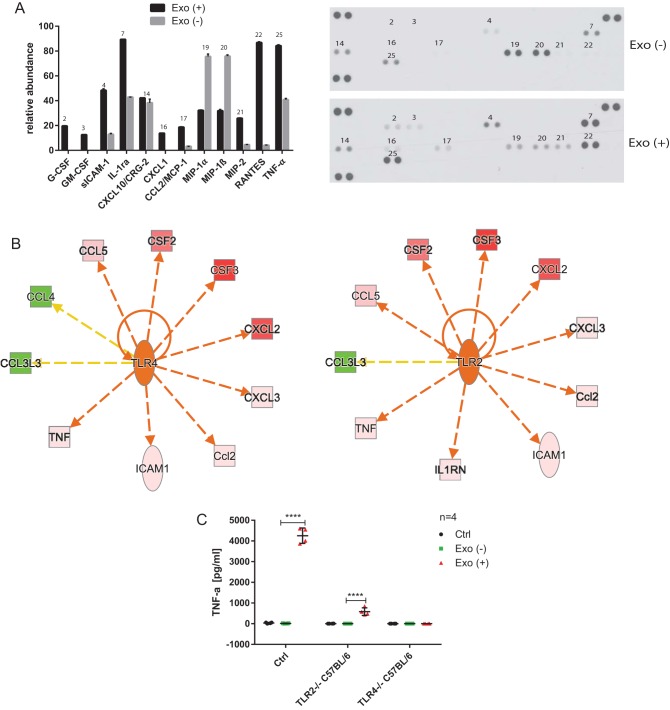
Exosomes elicit TLR4-dependent stimulation of TNF-α in naive macrophages. (A) CD9^+^ exosomes (fraction F10) isolated from *S*. Typhimurium-infected [2 hpi; Exo (+)] or uninfected [Exo (−)] RAW 264.7 macrophages (see Fig. 7A and B) were used to treat naive RAW 264.7 macrophages. After 24 h, the cell culture supernatants were collected, and 40 cytokines were analyzed by a proteome profiler mouse cytokine array kit, panel A (R&D Systems). The experiment was performed in two biological and two technical replicates. The pixel intensity of spots was measured by ImageJ, and the relative abundance was adjusted to the background and visualized as a graph. The numbers above the bars (left) correspond to the numbers above the spots on the membrane (right) for technical duplicates of cytokines. (B) Relative abundance data from the cytokine panel were imported into Ingenuity Pathway Analysis software to identify activated/inhibited cellular receptors. TLR2, TLR4, and TLR5 had the highest probability of being activated by the exosomes derived from infected macrophages (data for TLR2 and TLR4 are shown). Downregulation of MIP-1α (CCL3L3) and MIP-1β (CCL4) in cells treated with exosomes derived from infected versus uninfected cells was inconsistent with the IPA analysis. Abbreviations: TNF, TNF-α; ICAM1, sICAM-1; Ccl2, MCP-1; CXCL3, MIP-2; CXCL2, MIP-1; CSF3, G-CSF; CSF2, GM-CSF; CCL5, RANTES; CCL4, MIP-1β; CCL3L3, MIP-1α; IL-1RN, IL1RA. Red and green molecules indicate increased and decreased abundances, respectively. The orange arrows and central molecules (TLR2 and TLR4) indicate activation, while the yellow arrows indicate findings inconsistent with the predicted activation of central molecules. (C) Control murine macrophages and TLR2^−/−^ and TLR4^−/−^ C57BL/6 macrophages were treated with exosomes derived from uninfected or infected (MOI of 5:1, 2 hpi) RAW 264.7 macrophages or with PBS (control). After 24 h of treatment, the concentration of TNF-α in CCS was measured by ELISA. The results of four biological replicates are shown. One-way ANOVA test with Tukey's multiple testing correction was used to establish statistical significance. *P* values are indicated as follows: *, *P* ≤ 0.05; **, *P* ≤ 0.01; ***, *P* ≤ 0.001; and ****, *P* ≤ 0.0001.

Next, we used a cytokine array to perform relative quantification of 40 cytokines in cell culture supernatants of naive macrophages treated for 24 h with F2 (CD63^+^) and F10 (CD9^+^) exosomes derived from infected or uninfected murine RAW 264.7 macrophages. In comparison to F10 exosomes derived from uninfected cells, F10 exosomes obtained from infected macrophages induced an increased secretion of several cytokines including RANTES, TNF-α, IL-1ra, MIP-2, CXCL1, MCP-1, sICAM-1, granulocyte-macrophage colony-stimulating factor (GM-CSF), and G-CSF, but a decreased secretion of MIP-1α and MIP-1β (Fig. 9A). F2 exosomes obtained from infected cells also increased the levels of RANTES, TNF-α, IL-1ra, sICAM-1, MCP-1, and MIP-2, as well as SDF-1, but they did not affect MIP-1α and MIP-1β release from cells treated with these exosomes (see Fig. S7B in the supplemental material). An increase in TNF-α release from naive cells treated with exosomes derived from infected cells versus uninfected cells was previously confirmed by ELISA (Fig. 7 and 8), which further validates these data. Next, the relative abundance data from the cytokine panel were imported into IPA software to identify activation or inhibition of upstream regulators (Fig. 9B and data not shown). This unbiased IPA analysis revealed that three Toll-like receptors (TLRs)—TLR2, TLR4, and TLR5—had the highest probability of being activated by the F10 exosomes derived from infected macrophages. These TLRs can recognize pathogen-associated molecular patterns (PAMPs), including LPS and flagellin, and TNF-α, along with several other cytokines, is predicted to be activated by all three TLRs. However, attenuated secretion of MIP-1α and MIP-1β is inconsistent with the upregulation of these TLRs, which warrants further studies.

Subsequently, we tested whether murine macrophages lacking TLR2 or TLR4 receptors have an attenuated response to exosomes derived from *S*. Typhimurium-infected cells in comparison to mouse macrophages possessing these receptors but treated with the same exosomes. Although exosomes derived from infected macrophages led to a significant release of TNF-α in naive control cells, TLR4^−/−^ cells treated with these exosomes produced only a small amount of TNF-α compared to TLR4^−/−^ cells treated with exosomes produced by uninfected cells (Fig. 9C and see Fig. S11 in the supplemental material). Finally, TLR2^−/−^ cells produced a decreased but statistically significant amount of TNF-α. In summary, we show that TLR4 is necessary for exosome-induced TNF-α secretion in tested macrophages, although other TLRs, such as TLR2, might also be involved.

## DISCUSSION

Macrophages are specialized phagocytic cells maintaining metabolic homeostasis, and they possess distinct phenotypes depending on polarization (25). Activated M1 macrophages facilitate the host defenses against bacterial and viral pathogens, whereas M2-polarized macrophages promote wound healing and are anti-inflammatory. Macrophages secrete a wide variety of cytokines, which include interleukins (e.g., IL-6, IL-10, IL-12, and IL-1β) and chemokines (e.g., CXCL8, CXCL16, and CXCL10). These secreted proteins have profound effects on the host cell defense by regulating various aspects of cell signaling during infection, but they can also serve as biomarkers of cellular functions (26). Although the functions of secreted cytokines in host response to bacterial infections have been well studied (27), other proteins, such as cathepsins (28) and specific receptors (29), can also be secreted by macrophages. There have been several attempts to identify extracellular proteins of macrophages, which included mouse RAW 264.7 macrophages (29), human U937-derived macrophages (30), mouse BMDMs (31), human primary monocyte-derived macrophages (32), human primary macrophages (33), and human THP-1-derived macrophages (34). Extracellular proteomes of cells subjected to differentiation (30) or stimulation with LPS or IL-4 (31, 34) and influenza A virus-infected macrophages (33) were analyzed, but analysis of the extracellular proteome of human macrophages infected with bacteria has never been performed.

In this report, we describe a snapshot of an extracellular proteome of THP-1-derived human macrophages infected with *S*. Typhimurium for 90 min. A significant portion of the identified proteins was extracellular or exosomal, as expected (Fig. 1). A total of 90% of the proteins with significantly down- or upregulated extracellular abundance were annotated as present in extracellular vesicles or extracellular milieu (Fig. 1 and see Table S1 in the supplemental material). We identified several *S*. Typhimurium proteins, such as SipA (35), SipC (36), or phase 2 flagellin (Table 1), in the cell culture supernatants of infected cells. Identification of Salmonella effector proteins in cell culture supernatants of infected cells is not surprising, but it does support the validity of this proteomic study.

Protein ubiquitination was one of the top canonical pathways significantly altered by *S*. Typhimurium infection (Fig. 2 and 3). The extracellular levels of two proteins involved in this pathway, PSMA5 and OTUB1, were significantly increased in the cell culture media of infected macrophages. The extracellular presence of a proteasome subunit PSMA5 is intriguing since extracellular proteasomes have been previously identified (37), and their subunits possibly present in extracellular vesicles according to the Exocarta database (38), but they have not been studied in the context of infection. It is postulated that extracellular proteasomes could function in extracellular protein degradation (39, 40). Overall, the host ubiquitination pathway is widely involved in response to *S*. Typhimurium infection (9, 41, 42), but the involvement of ubiquitination in targeting proteins to exosomes during this infection is unknown. Another secreted protein of interest was OTUB1, the protein level of which was increased in cell culture medium of infected cells (Table 1 and Fig. 4). The extracellular presence of this enzyme was unexpected because its amino acid sequence is devoid of a signal peptide. OTUB1 is a cellular deubiquitinase (DUB) which regulates actin cytoskeleton (11, 43) and p53 stabilization (44), as well as the NF-κB and mitogen-activated protein kinase signaling pathways (45). Increased OTUB1 expression leads to an increased uptake of Yersinia enterocolitica and Y. pseudotuberculosis into host cells (11). In our study, OTUB1 was secreted starting at 1 hpi onwards, but it was undetectable in the cell culture media of uninfected cells. This finding led us to believe that there might be other deubiquitinases present in the extracellular milieu, a possibility that was confirmed by ubiquitin-specific active site probe-based activity profiling (see Fig. S3 in the supplemental material). In the absence of *S*. Typhimurium, there were active DUBs in the extracellular space, but infection affected the presence or activity of several DUBs; for instance, it led to an increase in active OTUB1 secretion, based on its reactivity with the ubiquitin-specific probe. OTUB1 does not have an N-terminal secretory motif, and because of that we hypothesized that this enzyme might be released via exosomes. Indeed, cells treated with GW4869 neutral sphingomyelinase inhibitor, which interferes with exosome release (14), released a reduced amount of OTUB1 upon infection. Moreover, OTUB1 was not found in a vesicle-free fraction of cell culture supernatant, but it was explicitly present in exosomes (Fig. 5). These data indicate that OTUB1 is released by exosomes in an nSMase2-dependent pathway, which requires sphingolipid ceramide since GW4869 inhibits this particular biogenesis pathway of exosomes (46). However, it cannot be ruled out that OTUB1 is also packaged into exosomes by the ESCRT-dependent pathway. GW4869 inhibitor used at the current concentration (which is limited by its solubility in dimethyl sulfoxide [DMSO] solvent) led to only a partial reduction in CD63 and OTUB1 secretion.

Next, we performed an initial evaluation of the role of exosomes secreted upon *S*. Typhimurium 2 hpi. Exosomes derived from infected macrophages triggered a rapid release of proinflammatory TNF-α in RAW 264.7 macrophages and BMDMs exposed to these exosomes. In contrast, an equal dose of exosomes derived from uninfected macrophages did not affect TNF-α release. This effect was dose dependent (Fig. 7A and B), and it also depended on the time of infection used to generate these exosomes (Fig. 7B). It has been shown that EVs produced by macrophages infected for 3 days with *S*. Typhimurium and Mycobacterium bovis provoke an increase in TNF-α production in uninfected macrophages, but other cytokines or earlier time points of infection were not tested for *S*. Typhimurium (18). In the case of exosomes released from M. bovis-infected macrophages, a minimum of 2 days of infection was needed to obtain exosomes effective in inducing TNF-α in naive cells (18). In our study, we used notably earlier times of infection (2 and 36 h), and exosomes produced under all of these conditions had a significant effect on TNF-α stimulation. Furthermore, exosomes derived from infected primary DCs did not stimulate a significant amount of TNF-α in either DCs or BMDMs, although DC-derived exosomes stimulated the release of chemokines such as CXCL10 or CXCL1 in naive DCs (Fig. 7D; see also Fig. S7A in the supplemental material). In contrast, BMDM-derived exosomes stimulated a significant TNF-α release in both naive DCs and BMDMs (Fig. 7C and D). These findings suggest that the proinflammatory function of exosomes depends on the parent cell. It is possible that biological cargo of DC-derived exosomes differs from BMDM-derived exosomes or that DC-derived exosomes are differentially internalized by the target cells, which should be addressed in future studies.

The function of exosomes in stimulation of the innate immune responses is complicated by the fact that there are multiple subpopulations of exosomes (23). We show that CD9^+^ (F10) exosomes of a higher density led to a more pronounced release of TNF-α in naive RAW 264.7 macrophages than CD63^+^ (F2) exosomes of a lower density (Fig. 8E). F10 exosomes derived from infected macrophages also led to an elongation of BMDMs (see Fig. S10). Cytokine analysis revealed that F10 exosomes obtained from infected macrophages induced an increased secretion of several cytokines (including RANTES, TNF-α, IL-1ra, MIP-2, CXCL1, MCP-1, sICAM-1, GM-CSF, and G-CSF) but a decreased secretion of MIP-1α and MIP-1β (Fig. 9A). F2 exosomes induced a release of RANTES, TNF-α, IL-1ra, sICAM-1, MCP-1, and MIP-2 similarly as F10 exosomes, but they additionally induced SDF-1 and did not affect MIP-1α and MIP-1β release (see Fig. S7B in the supplemental material). It is unclear whether exosomes affect the polarization of murine macrophages, but based on our data it is likely that they lead to a unique elongated phenotype associated with an increased secretion of IL-1ra, TNF-α, RANTES, and other cytokines (Fig. 9; see also Fig. S10 in the supplemental material).

LPS has been previously suggested to be transported via the secretory pathway of macrophages upon *S*. Typhimurium infection (18). We show that exosomes derived from macrophages infected with *S*. Typhimurium producing 1-monophosphorylated LPS triggered significantly less TNF-α in naive cells than did exosomes derived from macrophages infected with wild-type *S*. Typhimurium (Fig. 7E). However, these exosomes still led to a significant release of TNF-α compared to exosomes derived from uninfected cells. Since LPS toxicity was relevant in the production of proinflammatory exosomes, we tested whether the TLR4 signaling pathway is necessary for the function of exosomes in naive cells. Exosomes derived from macrophages infected with wild-type *S*. Typhimurium did not cause a significant release of TNF-α in TLR4^−/−^ cells (Fig. 9C; see also Fig. S11 in the supplemental material). Moreover, proteinase K treatment of exosomes significantly abolished TNF-α release in treated cells (see Fig. S8B in the supplemental material), whereas polymyxin B treatment did not completely abolish these proinflammatory effects of exosomes, but it reduced them significantly (see Fig. S8A in the supplemental material). Also, if exosomes were lysed by heat and their contents directly applied on naive macrophages, it led to a significant increase in TNF-α release compared to when the intact exosomes were used (see Fig. S8B in the supplemental material). All of these pieces of evidence suggest that LPS might be encapsulated and trafficked via exosomes (see Fig. S8 in the supplemental material), (18), but there might also be additional PAMPs contributing to the proinflammatory function of exosomes.

In summary, our study suggests that proinflammatory exosomes produced by macrophages infected with *S*. Typhimurium carry cargo, which is capable of stimulation of cytokine release from naive cells. We claim that LPS is enclosed within host exosomes and that this LPS stimulates a TLR4 signaling pathway in naive macrophages upon release. However, the protein cargo of exosomes sensitive to proteinase K is also relevant in triggering TNF-α release from naive cells, and it might lead to the stimulation of other immune receptors. Finally, we show that these proinflammatory exosomes not only induce a release of TNF-α but stimulate other cytokines, including TNF-α, RANTES, IL-1ra, MIP-2, CXCL1, MCP-1, sICAM-1, GM-CSF, and G-CSF. Because of the unique properties of exosomes, there is a therapeutic potential for these vesicles in immune surveillance (47) since they can be used for tissue-specific delivery of molecules (48, 49), which could include PAMPs. Future studies will reveal whether exosomes can be used *in vivo* for the cell-to-cell communication to induce the innate and adaptive host defenses, thus facilitating the clearance of bacterial infection.

## MATERIALS AND METHODS

### Reagents.

Chemicals were purchased from Fisher Scientific unless indicated otherwise. The following antibodies were used: mouse anti-β-actin antibody (Sigma-Aldrich, USA), mouse anti-HA antibody (Sigma-Aldrich), rabbit anti-OTUB1 antibody (Sigma-Aldrich) (10), mouse anti-GAPDH (Sigma-Aldrich), and rabbit anti-LTA4H and anti-PSMA5 (Cell Signaling, USA), as well as rabbit anti-CD9, anti-CD63, and anti-HSP70 antibodies (System Biosciences, USA).

### Cell culture.

THP-1 cells (ATCC TIB-202, American Type Culture Collection) were cultured in RPMI 1640 (Gibco/Life Technologies, Inc., USA) supplemented with 10% fetal bovine serum (FBS), 2 mM GlutaMAX (Gibco/Life Technologies) and 100 μg/ml penicillin-streptomycin (Life Technologies) in a humidified atmosphere of 5% CO_2_ at 37°C. For activation and differentiation of THP-1 cells, monocytes were incubated with 100 nM phorbol 12-myristate 13-acetate (PMA; Sigma-Aldrich) for 24 h. RAW 264.7 cells (ATCC TIB-71) were cultured in Dulbecco modified Eagle medium supplemented with 10% FBS and 100 μg/ml penicillin-streptomycin.

Bone marrow cells were harvested from the femurs and tibias of BALB/c mice to generate BMDMs and BMDCs. Bone marrow cells were cultured in bacterial petri dishes for 7 days in RPMI 1640 (Gibco/Life Technologies) containing 10% FBS and 100 μg/ml penicillin-streptomycin (Life Technologies). These media were supplemented with either 10 ng/ml GM-CSF (to generate BMDCs) or 10 ng/ml M-CSF (to generate BMDMs). The primary cells were replenished with fresh media containing GM-CSF or M-CSF on the fourth day of culture. Cells were stained with fluorochrome-conjugated anti-mouse immunoglobulin antibodies, including labeled mouse CD11b, CD11c, and MHC-II, and analyzed using a BD LSR Fortessa cell analyzer (BD Biosciences, USA). Populations of cells with >95% purity were used for further experiments.

*S*. Typhimurium wild-type ATCC 14028 (a gift from David Holden [50]), wild-type UK-1 (χ3761; a gift from Roy Curtiss III), and mutant UK-1 (Δ*pagL7* Δ*pagP81*::Plpp *lpxE* Δ*lpxR9* UK-1; χ9705, produces 1-monophosphoryl lipid A, in the background of χ3761; a generous gift from Roy Curtiss III [17]) were cultured in Luria-Bertani (LB) medium at 37°C and 200 rpm (MaxQ 6000 incubator; Thermo Scientific, USA) for 18 h. Overnight cultures were split to achieve an optical density at 600 nm (OD_600_) of 0.05 and were cultured until early exponential phase (OD_600_ = 0.5).

### Infection.

Cells were washed with PBS and incubated in growth medium containing no FBS or antibiotics for 90 min before infection. *S*. Typhimurium 12023 (ATCC 14028), wild-type UK-1 (χ3761), or mutant UK-1 (Δ*pagL7* Δ*pagP81*::Plpp *lpxE* Δ*lpxR9* UK-1) (17) was grown as described above until reaching early exponential phase (OD_600_ = 0.5). Alternatively, bacteria at stationary phase were used for infection. In each case, bacteria were washed with PBS and used to infect cells at a multiplicity of infection (MOI) of 5:1 or 50:1 (as indicated on the figures) for the times indicated on figures. Cell culture supernatants and cells were then collected. Cold PBS was added to the cells, and the cells were removed by using a cell scraper and centrifuged for 10 min at 500 × *g* (4°C). The cell culture supernatant was centrifuged for 10 min at 500 × *g* (4°C) to remove cellular debris, followed by spinning for 10 min at 9,000 × *g* (4°C) to remove the bacteria. The uninfected control cells were cultured in the same way but without the addition of bacteria. For infection times of >2 h, 100 μg/ml gentamicin was used to kill extracellular bacteria, as described previously (41). As a control, bacteria were inactivated with paraformaldehyde before addition to cells. In this experiment, 1 ml of bacterial cells grown to an OD_600_ of 0.5 were washed with PBS, centrifuged for 5 min at 5,000 × *g*, resuspended in 500 μl of PBS, and inactivated by adding 500 μl of 4% paraformaldehyde in PBS for 5 min at room temperature. Inactivated bacteria were washed twice with PBS and resuspended in RPMI 1640. Successful inactivation of bacteria was confirmed by culture on an agar plate at 37°C overnight. For heat inactivation, bacteria were resuspended in PBS and heated at 70°C for 1 h, centrifuged at 5,000 × *g* for 5 min, resuspended in 1 ml of PBS, and used for infection. Successful inactivation of *S*. Typhimurium was confirmed by culture on an agar plate at 37°C overnight. The same MOIs were used for infection with stationary and exponential-phase cultures, as well as with inactivated bacteria.

### LPS treatment.

LPS treatment of THP-1 macrophages was performed by addition of 1 μg/ml LPS obtained from *S*. Typhimurium (Sigma-Aldrich) to RPMI 1640 media. The cells were subsequently incubated for 1 h in a humidified atmosphere of 5% CO_2_ at 37°C.

### Cytotoxicity assay.

A CellTox Green cytotoxicity assay (Promega) was used to monitor cytotoxicity in real time. A total of 15,000 THP-1 cells were plated in a black 96-well plate, and the cells were stimulated with 100 nM PMA for 24 h. The cells were then infected at MOIs of 5:1 or 50:1 for a total of 195 min. Control cells were left uninfected or lysed with provided lysis buffer (Promega). Cell lysis was performed to calculate the maximum cytotoxicity value. The assay was performed according to the manufacturer's instructions, and cell cytotoxicity was monitored every 15 min for 195 min with a Cytation 3 multifunctional plate reader (Bio-Tek, USA). The results were analyzed by Prism 6 software (v6.07; GraphPad, USA). Two-way analysis of variance (ANOVA) and the post hoc Dunnett test were used to compute significance and correct for multiple comparisons.

### Isolation of secreted proteins for proteomics.

THP-1 monocytic cells were seeded on 5-cm dishes (5 million cells/dish) and treated with 100 nM PMA for 24 h. At 90 min before infection, the cells were incubated with antibiotic-free and FBS-free media. Cells were infected with *S*. Typhimurium for 90 min at an MOI of 50:1. The cell culture supernatant was spun down at 500 × *g* for 10 min at 4°C to remove cells and then at 9,000 × *g* for 10 min at 4°C to remove bacteria and debris. Samples were concentrated by using 3,000-Da cutoff Amicon centrifugal filters (Millipore, USA) until the volume reached 500 μl. The protein concentration was measured by using the modified Lowry method (DC kit; Bio-Rad). Samples containing 200 μg of protein were precipitated by using chloroform and methanol as described earlier (41). Protein samples were reduced and alkylated, followed by tryptic digestion as previously described (41). Peptides were desalted by using C_18_ Sep-Pak cartridges (WAT020515; Waters, USA) and liquid chromatography-tandem mass spectrometry analysis was performed with an LTQ Orbitrap Velos mass spectrometer (Thermo Fisher Scientific) equipped with an Advion Nanomate electrospray source (Advion, USA). Peptides were eluted from a C_18_ precolumn (100 μm [inner diameter] by 2 cm; Thermo Fisher Scientific), followed by analysis on an analytical column (75 μm [inner diameter] by 10 cm; C_18_; Thermo Fisher Scientific) by using a 148-min acetonitrile gradient. This analysis was performed as described previously (51).

### Proteomic data analysis.

Tandem mass spectra were extracted, charge state deconvoluted, and deisotoped using Proteome Discoverer 1.3 (Thermo Fisher Scientific). Tandem mass spectrometry (MS/MS) samples were analyzed by using the SEQUEST algorithm (v1.3.0.339; Thermo Fisher). The protein database used for data bank searching contained common contaminants (the common Repository of Adventitious Proteins [cRAP]), Salmonella enterica serovar Typhimurium proteins, and human proteins (UniProt; total of 87,396 entries) assuming trypsin as the digestion enzyme. SEQUEST was searched with a fragment ion mass tolerance of 0.8 Da and a parent ion tolerance of 10.0 ppm. Oxidation of methionine and carbamidomethylation of cysteine were specified in SEQUEST as variable modifications. Scaffold (v4.4.5; Proteome Software, Inc., USA) was used to validate MS/MS-based peptide and protein identifications. SEQUEST identifications required delta Cn scores of at least 0.2 and XCorr scores of at least 1.2, 1.9, 2.3, and 2.6 for singly, doubly, triply, and quadruply charged peptides, respectively. Protein identifications were accepted if they could be established at >95.0% probability and contained at least two identified peptides. The reported peptide false discovery rate (FDR) was 0.03%, and the protein FDR was 0.2%. Protein probabilities were assigned by the Protein Prophet algorithm. Proteins that contained peptides which could not be differentiated based on the MS/MS analysis were grouped. Proteins sharing significant peptide evidence were also grouped. The weighted spectral count was used for protein quantification, in which peptides were normalized by Scaffold. A minimum value of 0.1 spectral count was used to calculate a ratio (the ratio was calculated from the spectral count of proteins from infected cells and the spectral count of proteins from uninfected cells), which was then converted into a fold change (Excel, v12.2.8; Microsoft Office, USA) for subsequent bioinformatics analysis. A Fisher exact test was used to calculate statistical significance, and a *P* value of <0.05 indicated proteins with statistically significant changes in abundance.

### GO annotation analysis.

A PANTHER overrepresentation test was used (released 21 March 2016) for Gene Ontology (GO) term analysis of all the identified proteins. The GO ontology database (released 20 May 2016) was utilized in this test, wherein both molecular functions and cellular components were analyzed using experimentally verified GO terms. A Homo sapiens gene list containing all genes in the database was used as a reference gene list for the fold enrichment analysis. The Bonferroni correction for multiple testing was used in each case. The top GO terms were chosen based on their statistical significance. The fold enrichment for each GO term is shown on a graph (Excel v12.2.8).

Differentially regulated proteins were analyzed by using SignalP (SignalP 4.1) and SecretomeP (SecretomeP 2.0a) software, both available via the Technical University of Denmark (http://www.cbs.dtu.dk). For the SecretomeP analysis, an NM score of 0.5 was used as a cutoff to determine *ab initio* whether the proteins with no apparent signal peptide present (as determined by SignalP analysis) are part of the secretory pathway. The Venn graph of the results was prepared by using Venn Diagram Plotter (v1.5) and Adobe Illustrator (version CS6).

### Pathway, function and network analysis of extracellular proteins.

Ingenuity Pathway Analysis software (IPA; version 24390178) was used for functional analysis of extracellular proteins with abundance altered upon *S*. Typhimurium infection. Canonical pathways were analyzed by using a right-tailed Fisher exact test. The Benjamini-Hochberg multiple testing correction was applied to show the most significant results. The calculated significance represents the probability of association of proteins with the canonical pathway by random chance alone, and the −log of this *P* value is shown on the *y* axis of each graph. For canonical pathways, the square points connected by a thin line represent a ratio, which indicates the number of genes in a pathway from the data set divided by the total number of genes in the pathway (i.e., a reference gene list). The top protein network was overlaid with the most significant canonical pathways.

### Western blotting.

Protein samples were separated by using 4 to 12% gradient sodium dodecyl sulfate-polyacrylamide gel electrophoresis (SDS-PAGE) and transferred onto a polyvinylidene difluoride membrane by using Western blotting (Bio-Rad, USA), where transfer buffer contained 15% methanol, 25 mM Tris, and 192 mM glycine. Membranes were blocked with 5% nonfat milk, and proteins of interest were visualized by immunodetection using the appropriate antibodies and enhanced chemiluminescence.

### GW4869 inhibitor treatment.

THP-1 monocytic cells were seeded in a six-well plate (∼1.5 million cells/well) and treated with 100 nM PMA for 24 h. GW4869 (Cayman Scientific, USA) was dissolved in dimethyl sulfoxide (DMSO) to create a stock solution of 20 mM. Cells were incubated with antibiotic- and FBS-free medium supplemented either with 5 μM GW4869 or an equivalent volume of DMSO (vehicle control) 90 min before infection with *S*. Typhimurium (MOI of 50:1, 90-min infection). Cell culture supernatants and cell pellets were collected for Western blot analysis.

### Exosome isolation, transmission electron microscopy analysis, and Western blotting of vesicular proteins.

Exosomes derived from macrophages were isolated by ultracentrifugation (52). Briefly, after infection for the indicated times, cell culture supernatants containing secreted exosomes were collected and supplemented with PBS containing protease inhibitor cocktail (EDTA-free; Roche, USA). The samples were centrifuged sequentially under for 10 min at 500 × *g*, 10 min at 2,000 × *g*, and 40 min at 16,000 × *g* to remove cellular debris and bacteria. All of these steps were performed at 4°C. The supernatant was collected into a new vial and spun for 60 min at 100,000 × *g* at 4°C by using an SW41 Ti rotor (Beckman, USA). The supernatants were removed, the pellets containing exosomes were washed with cold PBS, and additional centrifugation was carried out at 100,000 × *g* for 60 min and at 4°C. The supernatant was removed, and the vesicles were resuspended in sterile PBS containing protease inhibitor cocktail.

For electron microscopy analysis, the vesicle pellet was resuspended in 4% paraformaldehyde in PBS. A sample containing 15 μl of resuspended vesicles was placed on Formvar and a carbon-coated grid. After 10 min of coating, a piece of filter paper was used to eliminate excess fluid from the grid, and a drop of 2% aqueous uranyl acetate was applied to the grid. After 20 s, the excess stain was removed with filter paper, and the grid was dried for 1 h before viewing by the JEOL JEM1230 transmission electron microscope at 80 kV (Mississippi State University). Alternatively, the protein samples prepared above (cell culture supernatant, vesicular fraction, and vesicle-free cell culture medium) were analyzed by SDS-PAGE and Western blotting.

For density-gradient separation, exosomes were purified by an OptiPrep (Axis-Shield, Oslo, Norway) discontinuous iodixanol gradient. The gradient was prepared by diluting a stock solution of OptiPrep (60%, wt/vol) with 0.25 M sucrose–10 mM Tris (pH 7.5) solution into 40, 20, 10, and 5% (wt/vol) iodixanol solutions. The density gradient was carefully loaded from the bottom to top with 3 ml of 40, 20, and 10% (wt/vol) iodixanol solutions, followed by 2.5 ml of 5% (wt/vol) iodixanol solution in Beckman Coulter centrifuge tubes (14 by 89 mm). A 500-μl sample of crude exosomes obtained by differential centrifugation was overlaid on the iodixanol gradient and centrifuged at 100,000 × *g* for 18 h at 4°C by using an SW41 Ti rotor (Sorvall). Then, 1-ml fractions were collected from the top to the bottom of the gradient and analyzed by using a NanoSight LM10 to establish the concentration, size, and intensity. Vesicles were diluted in PBS to reach a concentration of 1.0 × 10^8^ to 9.0 × 10^8^ particles/ml, and PBS used for dilutions was also measured by using NanoSight to ensure further that there was no contamination. Once the desired concentration of exosomes was reached, the sample was injected into the sample chamber of the NanoSight, and the particle size distribution was obtained through nanoparticle tracking analysis (NTA). NTA measures the mean square displacement of scattering species that cross the path of a sheet laser and calculates their hydrodynamic diameter using the Stokes-Einstein equation. Measurement of a large number of scatterers yields direct measurements of the hydrodynamic distribution and concentration of particulates in the sample. We verified that the fractions do not contain bacteria by culturing these fractions overnight on LB agar plates at 37°C.

### Treatment of macrophages with exosomes.

Crude exosome samples obtained via differential ultracentrifugation or subpopulations of exosomes purified by density gradient were used to treat naive cells. For these experiments, we established the protein concentration by lysing exosomes in 0.1% Rapigest (Waters) detergent in 100 mM ammonium bicarbonate (pH 7.5), followed by measurement of the amount of protein using a bicinchoninic acid (BCA) assay (Bio-Rad, USA). For treatments, we used 0.1 or 1 μg of intact exosomes derived from infected or uninfected macrophages (THP-1-derived human macrophages, RAW 264.7 mouse macrophages, primary mouse BMDMs or primary mouse BMDCs; see the figures for details). We tested two doses of exosomes (0.1 or 1 μg), two infection times of cells used to produce exosomes (2 and 36 h), and two different periods of exosome treatment (2 and 24 h).

Exosomes obtained from cell culture medium of *S*. Typhimurium-infected or uninfected cells (THP-1 macrophages, RAW 264.7 macrophages, BMDMs, or BMDCs; see the figures for details) were added to naive macrophages or DCs for 2 or 24 h at 37°C with 5% CO_2_. The supernatant was collected for ELISA analysis of TNF-α according to the manufacturer's instructions (Duo Set ELISA; R&D Systems, USA). Alternatively, a proteome profiler mouse cytokine array kit, panel A (R&D Systems), was used to obtain information about the relative levels of 40 cytokines, which was done in two technical and two biological replicates. The data were analyzed by GraphPad Prism (v6.07) by using the ordinary one-way ANOVA test along with Dunnett's multiple-comparison correction tests. The results obtained from each treatment sample were compared to the control sample (treatment with exosomes derived from uninfected cells) and PBS control, but other controls were also included in this comparison (see the figures for details). The multiplicity adjusted *P* value for each comparison was calculated.

Next, we determined whether exosomes activate Toll-like receptors (TLRs) in macrophages by using knockout murine cells. TLR4- or TLR2-deficient murine C57BL/6J macrophages (BEI Resources) were treated with 1 μg of exosomes derived from infected and uninfected murine macrophages for 24 h. The cell culture supernatant was collected, and TNF-α was quantified according to the manufacturer's instructions (Duo Set ELISA; R&D Systems).

## Supplementary Material

Supplemental material
