# Supplementary material for: Salmonella enterica Serovar Typhimurium Alters the Extracellular Proteome of Macrophages and Leads to the Production of Proinflammatory Exosomes
Source: Infect Immun. 2018 Jan 22;86(2):e00386-17. doi: 10.1128/IAI.00386-17 (PMC5778363; doi:10.1128/IAI.00386-17)
Supplement: Supplemental material [file supp_86_2_e00386-17__index.html]

Supplemental material 

# Salmonella enterica Serovar Typhimurium Alters the Extracellular Proteome of Macrophages and Leads to the Production of Proinflammatory Exosomes

## Supplemental material

- Supplemental file 1 -

  Supplemental methods. Fig. S1. Gentamicin protection assay. Fig. S2. OTUB1 transcript analysis in infected cells. Fig. S3. Probing activity of extracellular deubiquitinating enzymes by HA-Ub-VS. Fig. S4. NanoTracking analysis (NTA) of exosomes. Fig. S5. RAW 264.7-derived exosomes are internalized by naive RAW 264.7 cells. Fig. S6. TNF-α in macrophages treated with neutral sphingomyelinase inhibitor (GW4869) infected with *S.* Typhimurium. Fig. S7. Exosomes stimulate chemokine release in naive macrophages and DCs. Fig. S8. Endotoxin is a cargo of exosomes. Fig. S9. Subpopulations of THP-1 macrophage-derived exosomes from *S.* Typhimurium-infected cells trigger TNF-α and IL-1β release in uninfected THP-1 macrophages. Fig. S10. Cell morphology of naive BMDMs. Fig. S11. Concentration of TNF-α in CCS.

  PDF, 711K
- Supplemental file 2 -

  Table S1. Extracellular proteins of human macrophages with abundance altered upon *Salmonella enterica* serovar Typhimurium infection.

  XLSX, 219K
