# Supplementary material for: Salmonella enterica Serovar Typhimurium Alters the Extracellular Proteome of Macrophages and Leads to the Production of Proinflammatory Exosomes
Source: Infect Immun. 2018 Jan 22;86(2):e00386-17. doi: 10.1128/IAI.00386-17 (PMC5778363; doi:10.1128/IAI.00386-17)
Supplement: Supplemental material [file IAI.00386-17_zii999092286s1.pdf]

Supplementary Methods

Gentamicin protection assay

THP-1 monocytes were seeded onto a 12-well plate and differentiated into macrophages for 24 hours using 100 nM phorbol 12-myristate 13-acetate (PMA) in RPMI 1640 (Gibco, Life Technologies Inc., USA). 90 minutes before infection cells were washed with PBS and an incomplete RPMI 1640 medium was added onto cells. Cells were infected at a multiplicity of infection (MOI) of 50:1 using wild-type *Salmonella* Typhimurium (ATCC strain 12023) for 15 minutes, 90 minutes and 120 minutes. The cell culture supernatant was removed at each time point, and cells were washed twice with pre-warmed PBS. The cells were then supplemented with RPMI media 1640 containing 100 µg/ml of gentamicin and incubated for one hour. The cell culture supernatant was removed, and cells were washed twice with PBS. Buffer containing 0.1% Triton X-100 in PBS was placed on cells for 15 minutes, after which serial dilutions were plated on agar plates and incubated at 37°C for 16 hours. Cells were counted, and cell viability was measured by using an automatic cell counter (Bio-Rad, USA) before and infection to calculate the CFU/cell.

RT-PCR

Total RNA was isolated from uninfected and *S.* Typhimurium-infected THP-1 cells (MOI 50:1, 1.5 hpi) by using the Bio-Rad Aurum Total RNA Mini Kit following the manufacturer's instructions. '0 hour-control' represents cells before the 1.5-hour incubation, '1.5 hour- control' represents cells incubated for 1.5 hours in incomplete media, '1.5 hour – Infected' represents cells infected for 1.5 hours. Reverse transcription of 100 ng total RNA was completed using the iScriptTM cDNA Synthesis kit following manufacturer's instruction. The cDNA reactions were incubated at 25 °C for 5 minutes, 42°C for 30 minutes and 85°C for 5 minutes using the Bio-Rad CFX96 Real-Time System unit on the C1000 Touch Thermal Cycler. The nucleic acids were analyzed using the BioTek Take3 plate and analyzed for absorbance at 260 nm and 280 nm to calculate the purity of the nucleic acids. All the A260/A280 ratio for RNA and cDNA was ~2.0 and ~1.8, respectively. Quantitative PCR was completed with 400 ng of cDNA, iQ SYBR Green Supermix (Bio-Rad), OTUB1 and beta-actin forward and reverse primers (Bio-Rad) Prime PCR Assay. The optimized cycling protocol used for the complete qPCR samples were as following: initial denaturing was done at 95°C for 3 minutes, denaturing was done at 95°C for 15 seconds, and

annealing/extension was done at 55°C for 30 seconds. The denaturing and annealing/extension steps were completed for 40 cycles. OTUB1 transcripts (delta-delta Ct values) were quantified relative to beta-actin transcripts. Data (two technical replicates and three biological replicates) were analyzed in CFX Manager Software (v 3.1.1517.0823, Bio-Rad) and p-values were calculated.

Active-site probe labeling

Human influenza hemagglutinin (HA)-tagged ubiquitin vinyl sulfone ubiquitin-specific active-site probe (Ub-VS-HA) was used to analyze the activities of deubiquitinating enzymes (DUBs). THP-1 macrophages were infected (or not) for 0, 30, 60 and 90 minutes with *S*. Typhimurium as described above. Cell culture supernatant was collected and cell debris removed by centrifugation (500 × g for 10 minutes, followed by 9,000 x g for 10 min, where all steps were performed at 4°C). Sample containing equal amounts of protein were subjected to the reactions with Ub-VS-HA (Boston Biochem, USA) as we described previously (1, 2). The DUBs reacting with Ub-VS-HA probe were analyzed by SDS-PAGE, followed by Western blotting by using anti-HA antibody and OTUB1 antibody.

Silver stain

Silver stain was used to analyze SDS-PAGE containing CD63+ (F2) and CD9+ (F10) exosome samples from 4 µg of fractionated exosomes derived uninfected or *Salmonella*-infected RAW 264.7 macrophages and 0.1 µg LPS. The gel was fixed in 40% ethanol and 10% acetic acid for 1 hour. Next, the gel was washed with 30% ethanol for 20 minutes twice and subsequently washed with MilliQ water once for 20 minutes. For the silver reaction, the gel is then incubated in 0.02% sodium thiosulfate solution and then incubated with cold 0.1% Silver Nitrate solution containing 37% formalin for 20 minutes. After incubation, the gel is then washed in MiliQ water three times for 20 seconds each. The gel was developed solution containing 3% sodium carbonate with 37 % formalin and washed with miliQ water for 20 seconds. The development of the gel is stopped by washing the gel in 5% acetic acid for 10 minutes and washed with MilliQ water for 5 minutes.

Cell Treatment with LPS and Polymyxin B

RAW 264.7 macrophages were seeded in 24 well plates 24 hours before treatment. The following day the supernatant was aspirated and replaced with either DMEM complete media with or without 20 µg of polymyxin B for one hour before treatment with LPS or exosomes. LPS (500 pg or 1000 pg) and exosomes (1 µg) were used to treat cells containing complete media with or without polymyxin B for 24 hours. The supernatant was collected and spun at 800 x g to remove cells and subsequently analyzed for TNF-α using ELISA.

Cell Treatment of Exosomes with Proteinase K

Raw 264.7 macrophages were seeded in 24 well plates 24 hours before treatment. Exosomes were lysed at 98˚C for 10 minutes and then incubated in complete media containing 20 µg Proteinase K for 1 hour at 37˚C. The enzymatic activity of proteinase K was inhibited by adding 100 µM of phenylmethylsulfonyl fluoride (PMSF). The lysed or intact exosomes incubated with or without proteinase K were used to treat cells for 24 hours. The supernatant was collected and spun at 800 g to remove cells and subsequently analyzed for TNF-α using ELISA.

Limulus Amebocyte Lysate (LAL) Assay

LAL assay was used to quantify endotoxin in crude or fractionated exosomes derived from uninfected of *S.* Typhimurium-infected macrophages. A 96 well microplate was equilibrated at 37˚C for 10 minutes. Samples and standards were dispensed into the microplate and incubated at 37˚C for 5 minutes. Next, LAL solution was added to each well, and the plate was placed on a plate shaker for 10 seconds then incubated at 37 ˚C for 10 minutes. After that, a substrate solution is added to each well, mixed for 10 seconds using a plate shake and incubated at 37˚C for 6 minutes. Finally, stop reagent (25% acetic acid) is added to each well and mixed for 10 seconds. The assay is read using the Cytation3 plate reader (Biotek, USA) at 405 nm.

GW4869 Treatment of cells for ELISA assay

RAW 267.4 macrophages were seeded on 5 mm dishes 24 hours before treatment. Macrophages were incubated with 5 µM of GW4869 or the vehicle control containing DMSO one hour before infection. Exosomes derived from uninfected or infected macrophages with GW4869 or DMSO were isolated and used to treat naïve macrophage for 24 hours. The

supernatant was collected and spun at 800 x g to remove cells and subsequently analyzed for TNF-α using ELISA.

CFSE labeling of exosomes

RAW 264.7 mouse macrophages were infected with wild-type *S.* Typhimurium (UK-1; MOI 5:1) or left uninfected. Exosomes were collected from cell culture supernatant two hpi. Exosomes were then stained with ExoGreen carboxyfluorescein succinimidyl diacetate ester (CFSE) dye (SBI), after which the exosomes were precipitated by using ExoQuick (SBI) reagent and spun down at 21,000 x g and the pellet was resuspended in PBS, and their concentration was established by using a BCA protein assay. Naïve RAW 264.7 macrophages were plated on 6-well plates and treated with PBS (Ctrl) or with 0.1 µg ExoGreen-labeled exosomes derived from infected and uninfected macrophages. After 2 hours the cells were examined by fluorescent microscopy by using a GFP channel and contrast phase (EVOS Cell Imaging Systems, Thermo Fisher Scientific), and both images were merged. The cells with internalized exosomes were labeled with ExoGreen dye.

Confocal microscopy

BMDMs were cultured on coverslips and treated for 24 hours with F10 exosomes isolated from infected or uninfected RAW 264.7 macrophages. Cells were fixed and stained with rhodamine-phalloidin to visualize actin cytoskeleton. DAPI was used to stain nucleus, and differential interference contrast (DIC) was used to image cell morphology. Images were acquired by using Confocal Zeiss LSM800 microscope.

Supplementary Tables

Table S1. Extracellular proteins of human macrophages with abundance altered upon *Salmonella enterica* Typhimurium infection

Supplementary Figures

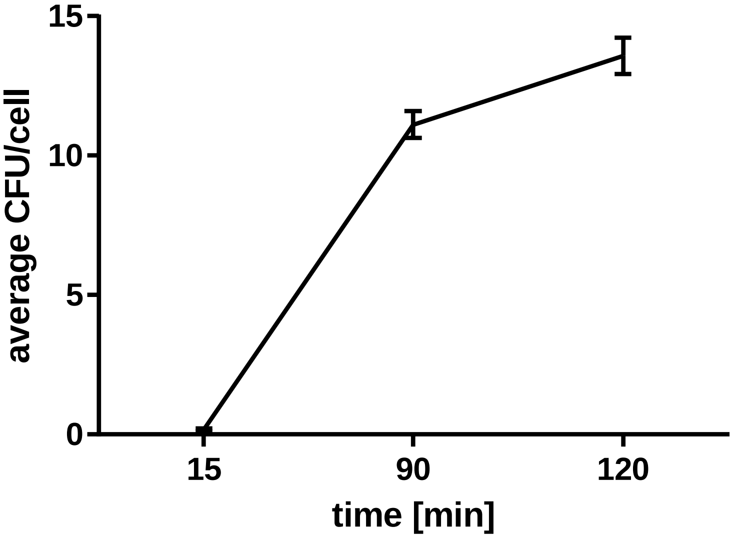

Figure S1. Gentamicin protection assay. THP-1 macrophages were infected at an MOI of 50:1 using wild-type *Salmonella* Typhimurium (ATCC strain 12023) for 15 minutes, 90 minutes and 120 minutes. The cell culture supernatant was removed at each time point, and cells were washed twice with pre-warmed PBS. The cells were then supplemented with RPMI media 1640 containing 100 µg/ml of gentamicin and incubated for one hour. The cell culture supernatant was removed, and cells were washed twice with PB, lysed with 0.1% Triton X-100 and dilutions plated on LB plate for enumeration of CFUs. Viable THP-1 cells were counted to calculate the CFU/cell.

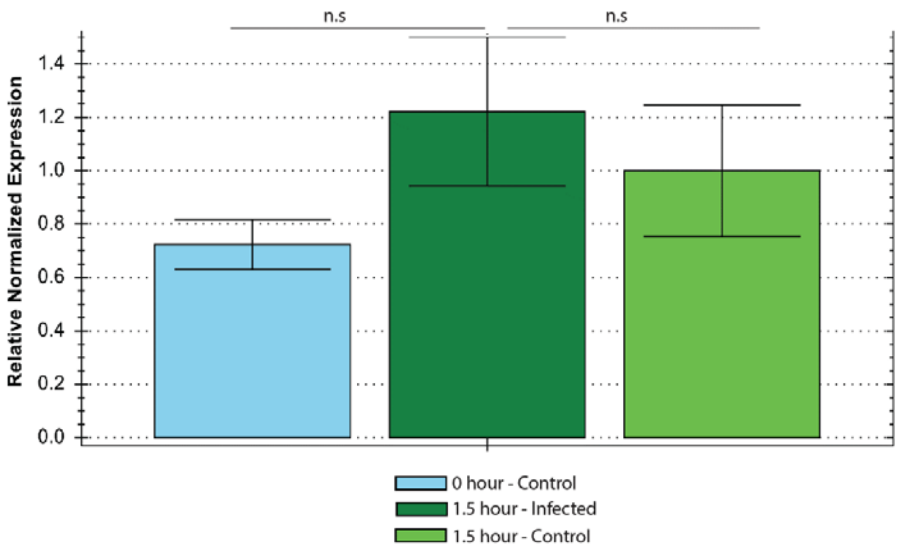

Figure S2. OTUB1 transcript analysis in infected cells. The OTUB1 transcript was quantified in uninfected and *S.* Typhimurium-infected THP-1 cells (MOI 50:1, 1.5 hpi) by using RT-PCR. '0 hour-control' represents cells before the 1.5-hour incubation, '1.5 hour- control' represents cells incubated for 1.5 hours in incomplete media, '1.5 hour – Infected' represents cells infected for 1.5 hours. OTUB1 transcripts (delta-delta Ct values) were quantified relative to beta-actin transcripts. Data (two technical replicates and three biological replicates) were analyzed in CFX Manager Software (v 3.1.1517.0823, Bio-Rad) and p-values were calculated. n.s., non-significant p-value.

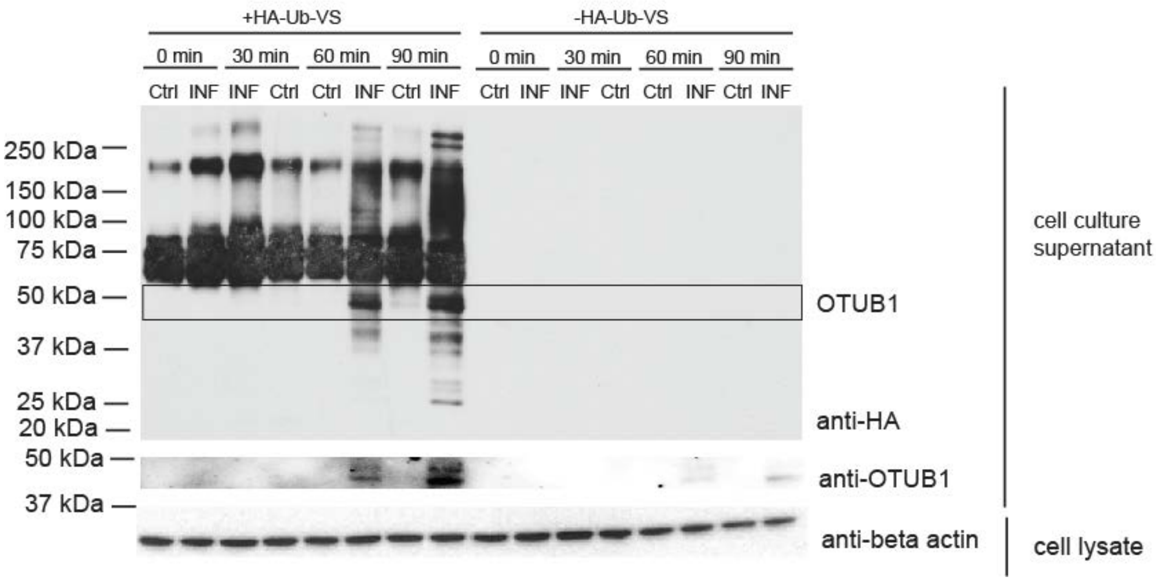

Figure S3. Probing activity of extracellular deubiquitinating enzymes by HA-Ub-VS. THP-1 macrophages were infected or left uninfected for 0, 30, 60 and 90 minutes with *S.* Typhimurium. Cell culture medium was collected at each time point in duplicates. Extracellular proteins were exposed to the reaction with the ubiquitin-specific active-site probe (HA-Ub-VS), which reacts with the active site of deubiquitinating enzymes. Deubiquitinating enzymes that reacted with the probe were detected by anti-HA Western blotting. OTUB1 was also visualized by anti-OTUB1 western blotting. Protein content in cell pellet has been analyzed by using anti-OTUB1 and anti-β-actin western blotting.

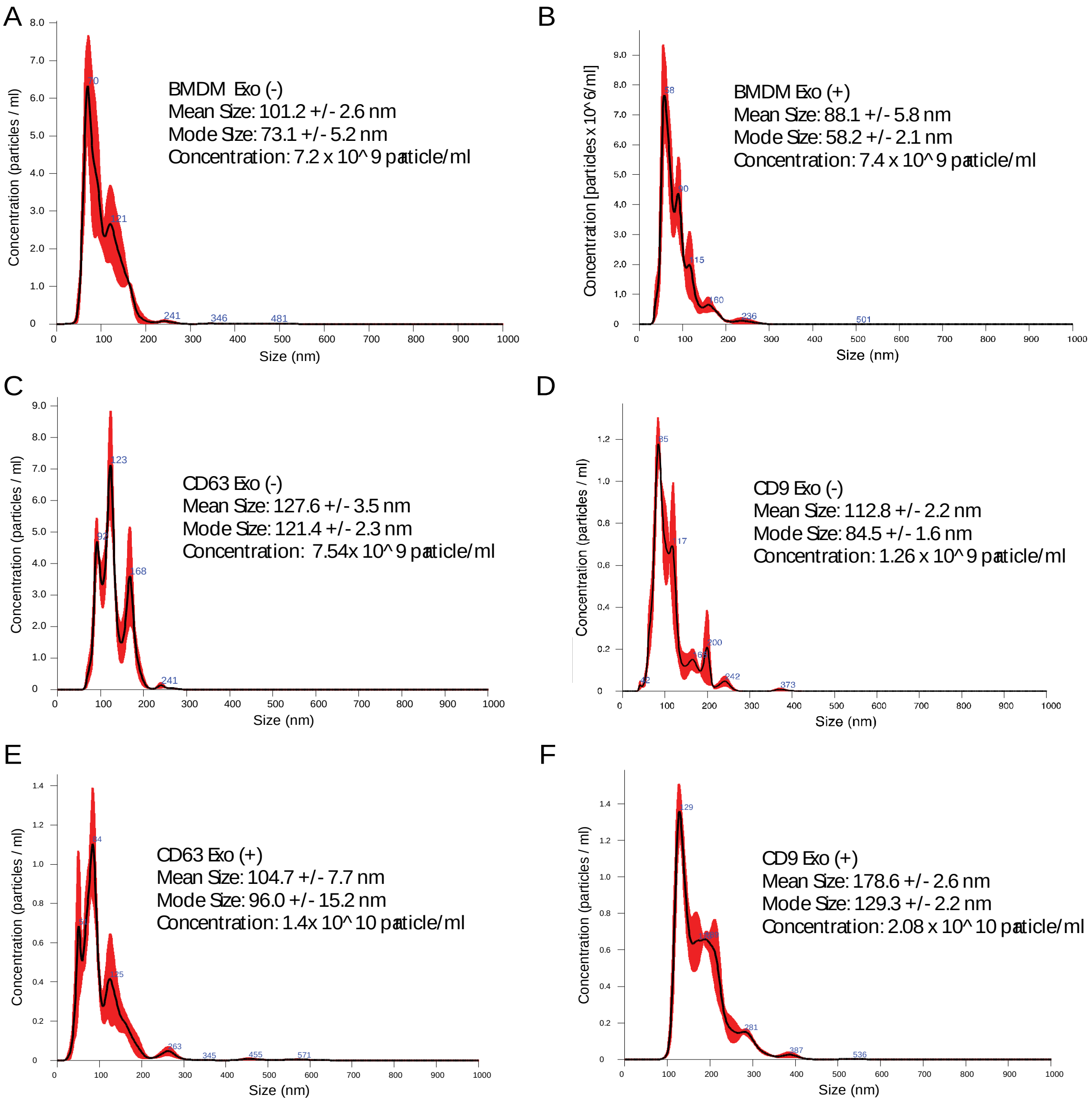

Figure S4. NanoTracking Analysis (NTA) of exosomes. NTA was used to determine the mean, mode and concentrations of exosomes derived from uninfected (A) and *S.* Typhimurium-infected (B) BMDMs. Exosomes derived from infected [(D) and (F)] and uninfected [(C) and (E)] THP-1 cells were fractionated by density gradient and NTA analysis of CD63-enriched [(C) and (E)] as well as CD9-enriched [(D) and (F)] was performed. In each case, exosome samples were diluted until ~10^8 particles/ml were detected by Nanosight. Dilution factor was calculated, and the concentration was reported on each graph. Exo(+), exosomes derived from infected macrophages; Exo(-), exosomes derived from uninfected macrophages.

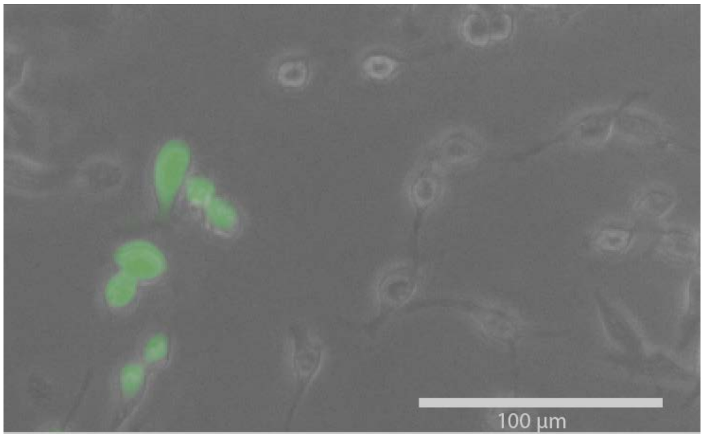

Figure S5. RAW 264.7-derived exosomes are internalized by naïve RAW 264.7 cells. RAW 264.7 mouse macrophages were infected with wild-type *S.* Typhimurium (UK-1; MOI 5:1) or left uninfected. Exosomes were collected from cell culture supernatant two hpi. Exosomes were then stained with ExoGreen carboxyfluorescein succinimidyl diacetate ester (CFSE) dye (SBI), after which the exosomes were precipitated by using ExoQuick (SBI) reagent and spun down at 21,000 x g and the pellet was resuspended in PBS, and their concentration was established by using a BCA protein assay. Naïve RAW 264.7 macrophages were plated on 6-well plates and treated with PBS (Ctrl) or with 0.1 µg ExoGreen-labeled exosomes derived from infected and uninfected macrophages. After 2 hours the cells were examined by fluorescent microscopy by using a GFP channel and contrast phase (EVOS Cell Imaging Systems, Thermo Fisher Scientific), and both images were merged. The cells with internalized exosomes were labeled with ExoGreen dye (as an example are shown RAW 264.7 macrophages treated with exosomes derived from infected 264.7 cells), but none of the cells treated with ExoGreen dye alone were labeled under these conditions (not shown).

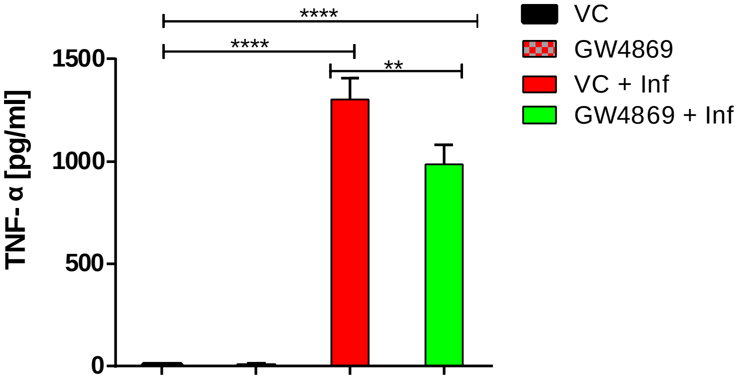

Figure S6. TNF-α in macrophages treated with neutral sphingomyelinase inhibitor (GW4869) infected with *S.* Typhimurium. RAW 264.7 macrophages were treated with GW4869 [5 µM] or an equal volume of DMSO, a vehicle control (VC), and subjected to infection with *S.* Typhimurium for 2 hours (Inf) or left uninfected. The cell culture supernatant was collected and analyzed for TNF-α. One-way ANOVA with Tukey's test was used to test for statistical significance. P-values were indicated as follows: * $p \leq 0.05$; ** $p \leq 0.01$; *** $p \leq 0.001$; **** $p \leq 0.0001$.

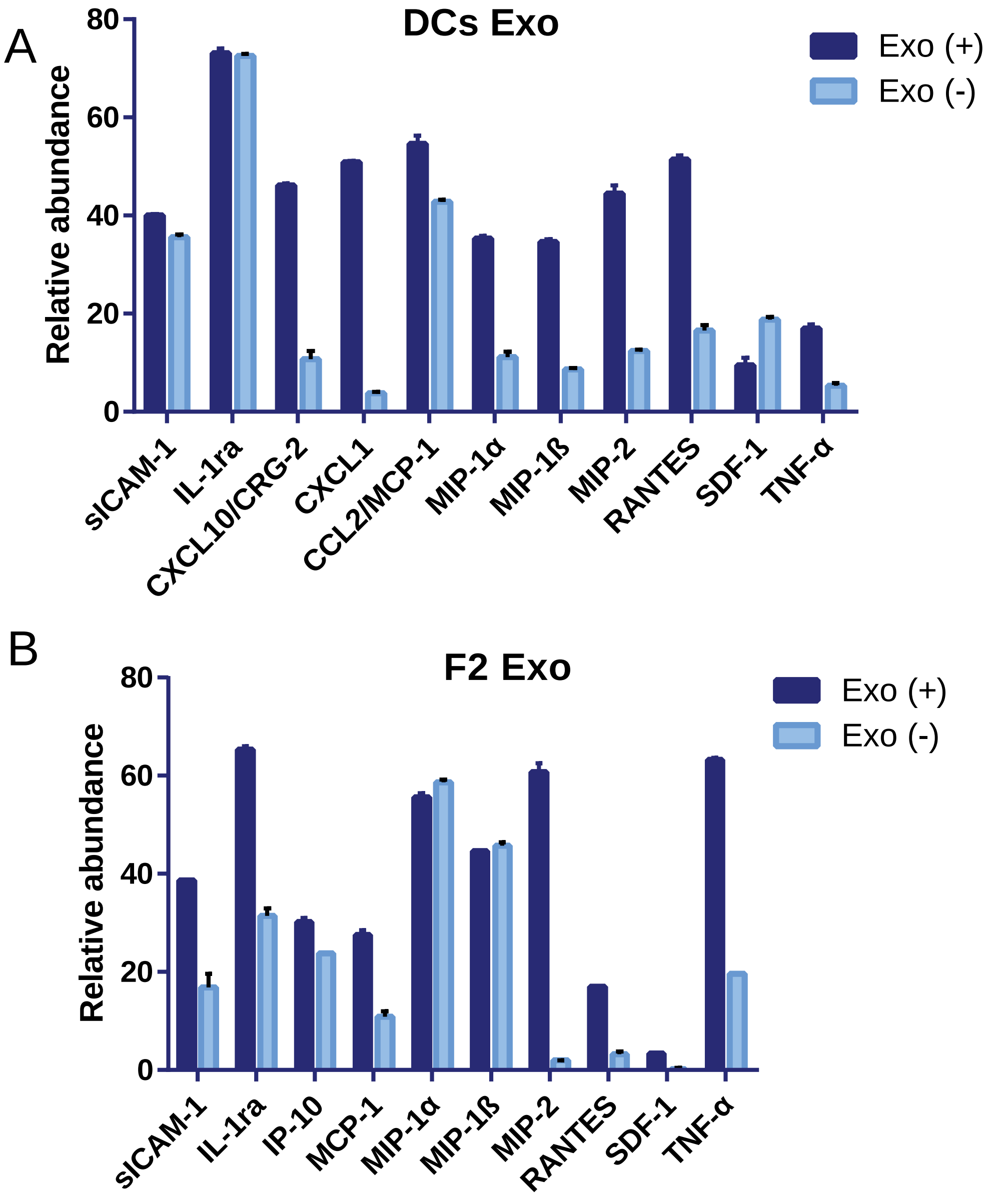

Figure S7. Exosomes stimulate chemokine release in naïve macrophages and DCs. (A). Exosomes produced from *S*. Typhimurium-infected [2 hpi; Exo (+)] and not infected [Exo (-)] murine bone marrow-derived DC (BMDCs) were used to treat naïve BMDCs for 24 hours. After 24 hours CCS was collected, and 40 chemokines were analyzed by Proteome Profiler Mouse Cytokine Array Kit, Panel A (R&D Systems, USA). The pixel intensity of spots was measured by ImageJ, the relative abundance was adjusted to the background and visualized as a graph. **(B).** CD63-positive exosomes (fraction F2) isolated from *S.* Typhimurium-infected (2 hpi) RAW 264.7 macrophages were used to treat naïve RAW 264.7 macrophages. Released chemokines were measured as in (A).

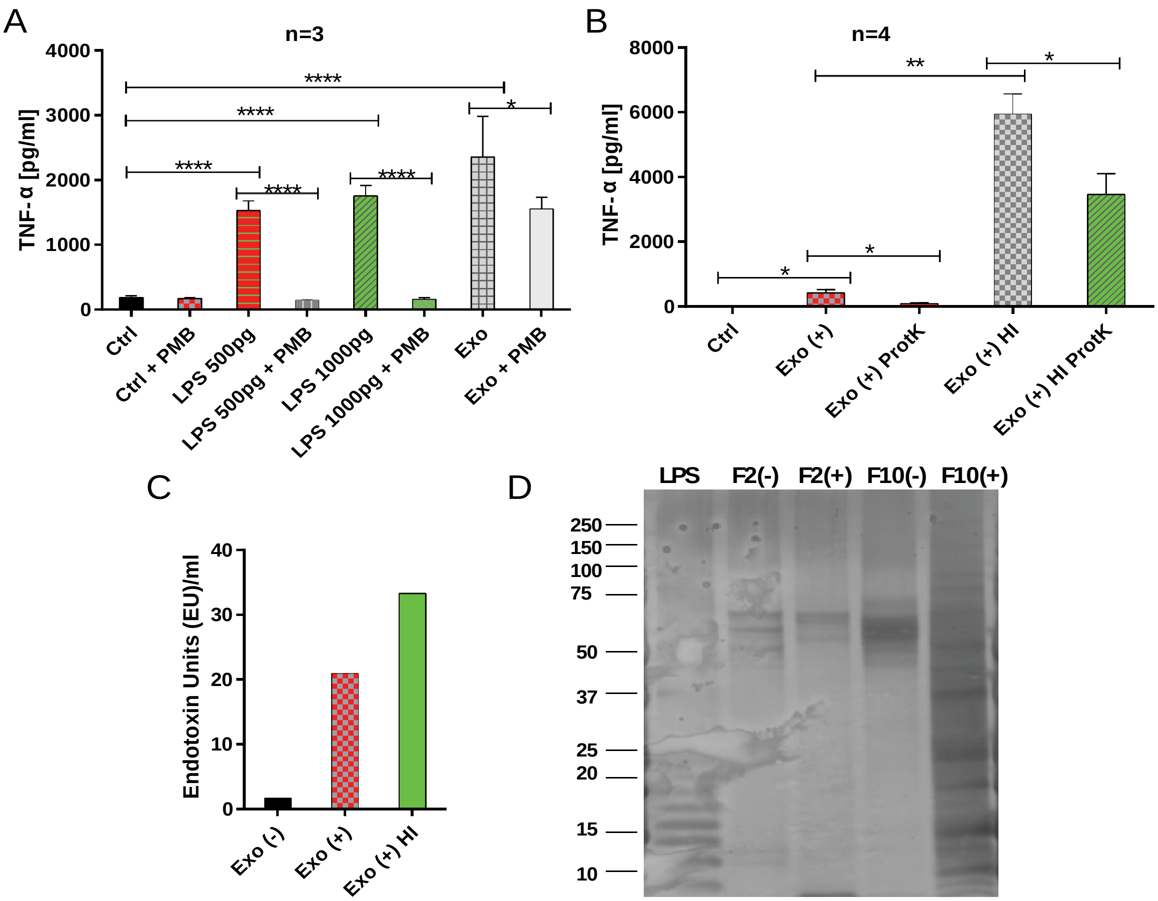

Figure S8. Endotoxin is a cargo of exosomes. (A) RAW 264.7 macrophages were treated with LPS (500 pg/ml and 1000 pg/ml) or exosomes (1 µg) derived from RAW 264.7 macrophages infected with *S.* Typhimurium (2 hpi) in presence or absence of 20 µg/ml of polymyxin B (PMB) for 24 hours. ELISA was used to quantify TNF-α release to cell culture supernatant. **(B)** Exosomes (1 µg) derived from *Salmonella*-infected macrophages were lysed or left intact and treated with or without Proteinase K (20 µg). RAW 264.7 cells were treated with equal amount of material for 24 hours, and ELISA was used to quantify release TNF-α. **(C).** LAL assay was used to quantify endotoxin in intact exosome preparations derived from uninfected macrophages as well as from intact and lysed (HI) exosomes derived from *Salmonella*-infected macrophages. Samples were analyzed in duplicate and a representative experiment is shown. **(D).** Protein and LPS components in F2 and F10 exosomes derived from uninfected and *Salmonella*-infected macrophages were analysed by SDS-PAGE and silver stain. As a control 0.1 µg LPS (*S.* Typhimurium) was used. Exo(+), exosomes derived from infected macrophages; Exo(-), exosomes derived from uninfected macrophages. One-way ANOVA with Tukey's test was used

to test for statistical significance for all figures. P-values were indicated as follows: * p≤ 0.05; ** p ≤ 0.01; *** p≤ 0.001; **** p ≤ 0.0001.

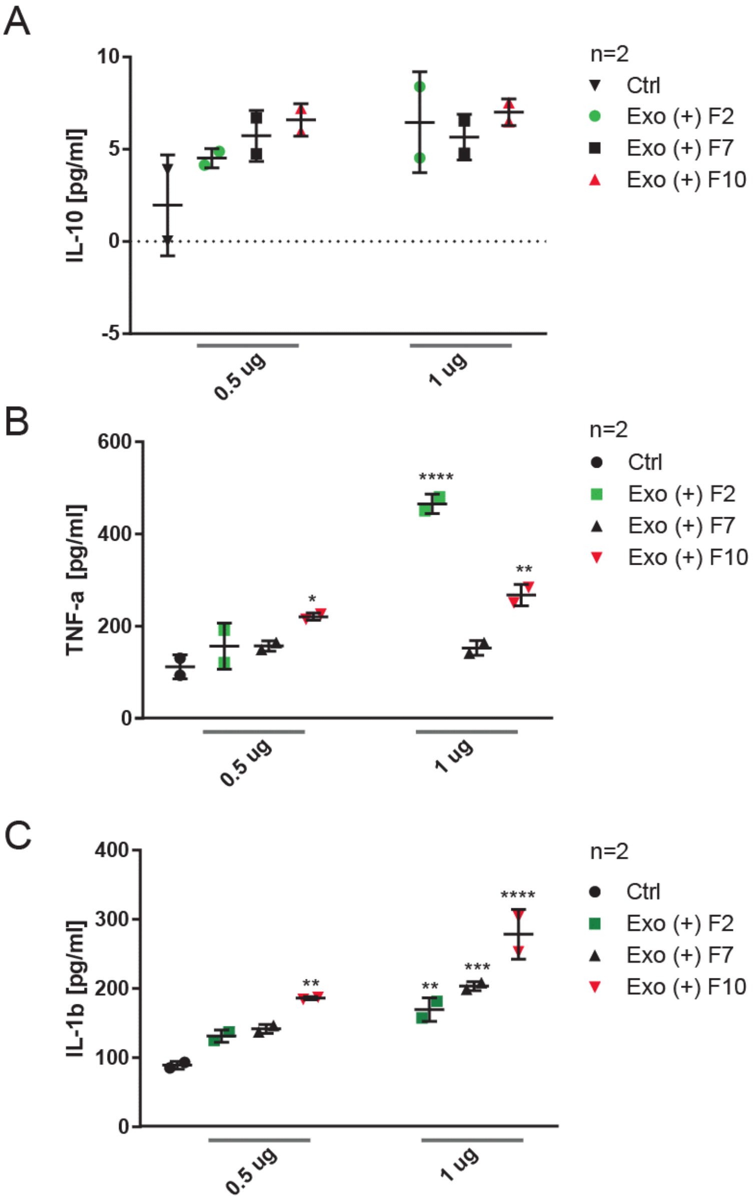

Figure S9. Subpopulations of THP-1 macrophage-derived exosomes from *S.* Typhimurium-infected cells trigger TNF-α and IL-1β release in uninfected THP-1 macrophages. PBS control (Ctrl) or 0.1 μg exosomes isolated from *S.* Typhimurium-infected (2 hpi) THP-1 macrophages (fractions F2, F7, and F10) were used to treat naïve THP-1 macrophages for 24 hours, after which released IL-10 (A), IL-1β (B) and TNF-α (C) were quantified by ELISA assays. Exo(+), exosomes derived from infected macrophages; Exo(-), exosomes derived from uninfected

macrophages. P values were indicated as follows: * p≤ 0.05; ** p ≤ 0.01; *** p≤ 0.001; **** p ≤ 0.0001.

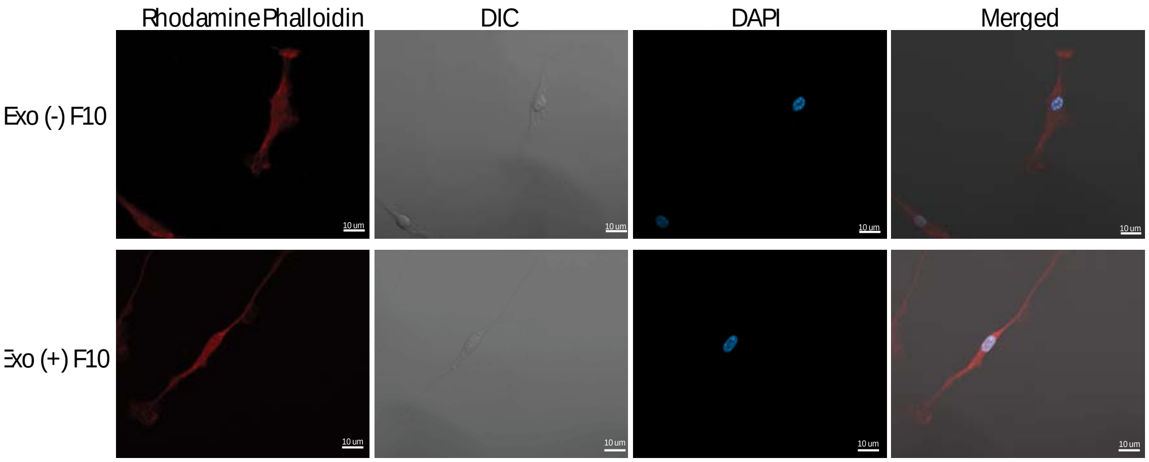

Figure S10. Murine BMDMs were cultured on coverslips and treated for 24 hours with F10 exosomes isolated from *S.* Typhimurium-infected (2 hpi) or uninfected RAW 264.7 macrophages (B). Cells were fixed and stained with rhodamine-phalloidin to visualize actin cytoskeleton. DAPI was used to stain nucleus, and differential interference contrast (DIC) was used to image cells.

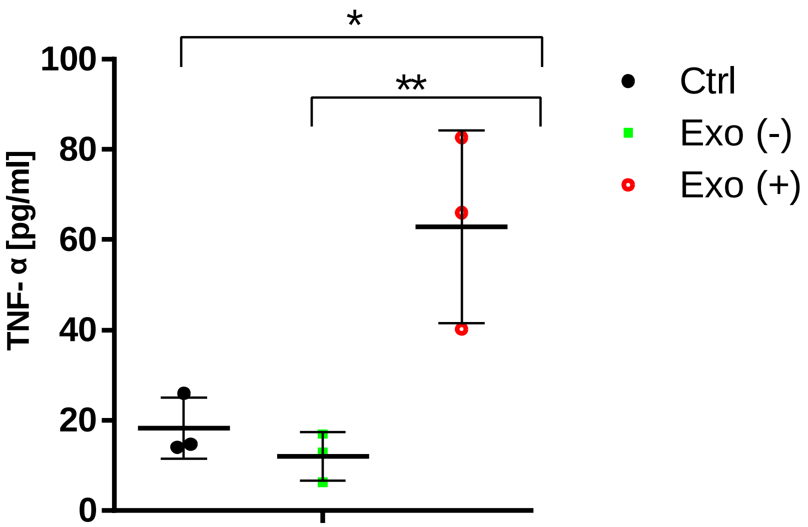

Figure S11. TLR 4-/- C57BL/6 macrophages were treated with exosomes derived from uninfected or infected (MOI 5:1, 2 hpi) RAW 264.7 macrophages or with PBS (control). After 24 hours of treatment concentration of TNF-α in CCS was measured by ELISA. Four biological replicates are shown. One-way ANOVA test with Tukey's multiple testing correction was used to establish statistical significance. P values were indicated as follows: * $p \leq 0.05$; ** $p \leq 0.01$; *** $p \leq 0.001$; **** $p \leq 0.0001$.

Supplemental Bibliography:

1. Edelmann MJ, Kramer HB, Altun M, Kessler BM. 2010. Post-translational modification of the deubiquitinating enzyme otubain 1 modulates active RhoA levels and susceptibility to Yersinia invasion. FEBS J 277:2515-30.
2. Kummari E, Alugubelly N, Hsu CY, Dong B, Nanduri B, Edelmann MJ. 2015. Activity-Based Proteomic Profiling of Deubiquitinating Enzymes in Salmonella-Infected Macrophages Leads to Identification of Putative Function of UCH-L5 in Inflammasome Regulation. PLoS One 10:e0135531.
